# Are fission–fusion dynamics consistent among populations? A large‐scale study with Cape buffalo

**DOI:** 10.1002/ece3.6608

**Published:** 2020-08-11

**Authors:** Elodie Wielgus, Daniel Cornélis, Michel de Garine‐Wichatitsky, Bradley Cain, Hervé Fritz, Eve Miguel, Hugo Valls‐Fox, Alexandre Caron, Simon Chamaillé‐Jammes

**Affiliations:** ^1^ Division of Biology and Conservation Ecology Manchester Metropolitan University Manchester UK; ^2^ LTSER France Zone Atelier “Hwange,” Hwange National Park, Bag 62, Dete Zimbabwe ‐ CNRS HERD (Hwange Environmental Research Development) Program Dete Zimbabwe; ^3^ CEFE CNRS Univ. Montpellier Univ. Paul Valéry Montpellier 3 EPHE IRD Montpellier France; ^4^ ASTRE CIRAD INRA Université de Montpellier Montpellier France; ^5^ Forêts et Sociétés CIRAD Montpellier France; ^6^ Forêts et Sociétés Université de Montpellier CIRAD Montpellier France; ^7^ CIRAD UMR ASTRE Bangkok Thailand; ^8^ Faculty of Veterinary Medicine Kasetsart University Bangkok Thailand; ^9^ Laboratoire de Biométrie et Biologie Evolutive UMR 5558 Centre National de la Recherche Scientifique (CNRS) Université Claude Bernard Lyon 1 Villeurbanne Cedex France; ^10^ MIVEGEC, IRD CNRS Université de Montpellier CNRS Montpellier France; ^11^ SELMET Université de Montpellier CIRAD INRA Montpellier Sup. Agro Montpellier France; ^12^ Faculdade de Veterinária Universidade Eduardo Mondlane Maputo Mozambique; ^13^ Department of Zoology & Entomology Mammal Research Institute University of Pretoria Pretoria South Africa

**Keywords:** association patterns, dyadic interactions, home range overlap, multi‐population, seasonality

## Abstract

Fission–fusion dynamics allow animals to manage costs and benefits of group living by adjusting group size. The degree of intraspecific variation in fission–fusion dynamics across the geographical range is poorly known. During 2008–2016, 38 adult female Cape buffalo were equipped with GPS collars in three populations located in different protected areas (Gonarezhou National Park and Hwange National Park, Zimbabwe; Kruger National Park, South Africa) to investigate the patterns and environmental drivers of fission–fusion dynamics among populations. We estimated home range overlap and fission and fusion events between Cape buffalo dyads. We investigated the temporal dynamics of both events at daily and seasonal scales and examined the influence of habitat and distance to water on event location. Fission–fusion dynamics were generally consistent across populations: Fission and fusion periods lasted on average between less than one day and three days. However, we found seasonal differences in the underlying patterns of fission and fusion, which point out the likely influence of resource availability and distribution in time on group dynamics: During the wet season, Cape buffalo split and associated more frequently and were in the same or in a different subgroup for shorter periods. Cape buffalo subgroups were more likely to merge than to split in open areas located near water, but overall vegetation and distance to water were very poor predictors of where fission and fusion events occurred. This study is one of the first to quantify fission–fusion dynamics in a single species across several populations with a common methodology, thus robustly questioning the behavioral flexibility of fission–fusion dynamics among environments.

## Graphical abstract

The frequency at which Cape buffalos split and join allows them to adjust to environmental conditions. In 3 distinct populations, the associations between individuals last on average between 1 and 3 days, but seasonal differences exist: In the dry season, buffalos split and join less frequently but stay in the same subgroup for longer periods than in wet season. Vegetation openness and distance to water are poor predictors of where fission and fusion events occur.

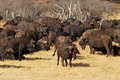

## 1 INTRODUCTION

Identifying the factors that drive social organization is central to understanding the ecology and evolution of animal populations. Animal social organizations range from solitary, where individuals meet occasionally and for mating during the breeding season, to systems whereby animals live in stable groups with individuals remaining together over several years (Clutton‐Brock, 2016). Groups can also be much more fluid, with regular splitting (i.e., fission) and merging (i.e., fusion) of subgroups, and the degree of fission–fusion dynamics between group members can be seen as a characteristic of any social system (Aureli et al., 2008).

Moderate to high levels of fission–fusion dynamics have been reported in a range of taxa (primates: Lehmann & Boesch, 2004, large mammalian herbivores: Archie, Moss, & Alberts, 2006, Fortin et al., 2009, Bercovitch & Berry, 2010, macropods: Best, Seddon, Dwyer, & Goldizen, 2013, cetaceans: Connor, Wells, Mann, & Read, 2000, fish: Kelley, Morrell, Inskip, Krause, & Croft, 2011, bats: Kerth & König, 1999). Decisions to split or merge are thought to be related to spatial and temporal variation in the costs and benefits of grouping, *for example,* changes in resource availability, competition (Chapman, 1990; Pépin & Gerard, 2008), predation risk (e.g., through habitat structure, Hill & Lee, 1998, Fortin et al., 2009), activity synchronization (Conradt & Roper, 2000), or in the risk of disease or pathogen transmission (Kashima, Ohtsuki, & Satake, 2013).

Most studies on fission–fusion dynamics published to date have either focused on describing dynamics in a single population (e.g., Lehmann & Boesch, 2004) or comparing fission–fusion dynamics in populations of different species living in the same area (e.g., Parra, Corkeron, & Arnold, 2011). Little is known about the variability of fission–fusion dynamics among populations of a given species (e.g., Kelley et al., 2011). As heterogeneity in the environment across the geographical ranges of a species can influence social behavior (Baden, Webster, & Kamilar, 2016), fission–fusion dynamics may vary between populations. Comparing fission–fusion dynamics from several populations located in different areas would provide insight into the behavioral flexibility of a species to adjust to heterogeneous environmental constraints. Standardized comparative studies would also allow a better determination of the factors influencing fission–fusion dynamics at the species level.

**Figure 1:**
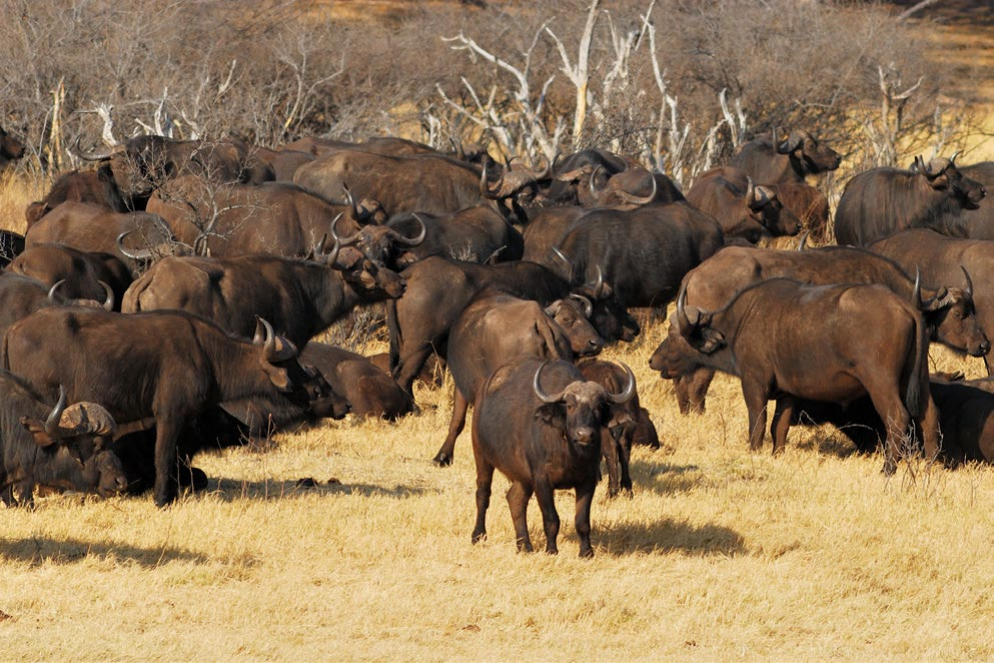
A subgroup of Cape buffalo (mainly females) near Hwange National Park, Zimbabwe. Photo © Stéphanie Périquet

Cape buffalo (*Syncerus caffer caffer*) lives in large (up to 1,500 individuals) mixed‐sex groups, primarily females and their offspring, subadults of both sexes, and a smaller proportion of adult males (Figure 1). Each group occupies a home range that overlaps very little with other groups (Prins, 1996; Sinclair, 1977, Wielgus et al. in prep). Within these large groups, subgroups of individuals split and merge regularly. The critical characteristics of these so‐called fission–fusion patterns, such as duration of subgroup splitting, and merging remain mostly unknown (but see Bennitt, Bonyongo, & Harris, 2018). The factors which appear to drive group dynamics in Cape buffalo remain unclear with conflicting results from different studies. In Chobe National Park (Botswana), Cape buffalo formed larger subgroups during the dry season, when resources are more limited (Halley, Vandewalle, Mari, & Taolo, 2002) but the opposite was reported in Serengeti National Park (Tanzania, Sinclair, 1977) and Klaserie Private Nature Reserve (South Africa, Ryan, Knechtel, & Getz, 2006). In Lake Manyara National Park (Tanzania), Cape buffalo groups tended to exhibit fission–fusion patterns strongly related to group size: Large groups split more frequently than smaller ones (Prins, 1989). Finally, changes in Cape buffalo group dynamics living in Addo Elephant National Park (South Africa) are related to predation, with buffalos aggregating into larger subgroups following the reintroduction of lions into the park (Tambling et al., 2012).

In order to better understand the patterns and drivers of fission and fusion in Cape buffalo, we employed a comparative approach incorporating data collected over wet and dry seasons across three distinct populations living in similar environmental conditions (Gonarezhou National Park and Hwange National Park, Zimbabwe; Kruger National Park, South Africa). Much of the previous research on fission–fusion dynamics in Cape buffalo, and generally on other species, is based on the observation of how the size and composition of subgroups change over time (Aureli et al., 2008; Prins, 1996). In this study, we take a different approach by studying fission–fusion dynamics at the individual level, using GPS tracking data. This approach is increasingly used (e.g., Loretto et al., 2017, Lesmerises, Johnson, & St‐Laurent, 2018, for Cape buffalo see Bennitt et al., 2018) as it provides detailed information on when and where individuals are in the same subgroup but does not provide information on subgroup size and composition. We used GPS tracking data to quantify the time that pairs of Cape buffalo (dyads) spent in the same subgroup and the temporal dynamics of fission and fusion events. We explored seasonal changes in fission–fusion dynamics. Building on our knowledge of the species’ ecology and on consistent results from previous studies, we also specifically tested the predictions that (a) fission and fusion events would occur more during the periods when Cape buffalos are more active, that is, early in the morning and late afternoon, because conflicts of interest in the upcoming activities or directions would be higher (Cornélis et al., 2011; Valls‐Fox et al., 2018); (b) Cape buffalo subgroups would be more likely to meet (fusion event) and remain together in open habitats, as large herbivores are commonly found in large groups in open habitats where visibility is higher (Isvaran, 2007; Jarman, 1974; Pays, Benhamou, Helder, & Gerard, 2007), facilitating social cohesion, reducing predation risk against ambush predators and, possibly for grazers, where forage is more abundant; and (c) the scarcity of water during the dry season would increase the probability that subgroups meet, and remain together for some time, near water points. We reveal new insights into Cape buffalo fission–fusion dynamics and provide one of the first studies demonstrating the consistency of fission–fusion dynamics across populations.

## 2 MATERIALS AND METHODS

### 2.1 Study areas

**Figure 2:**
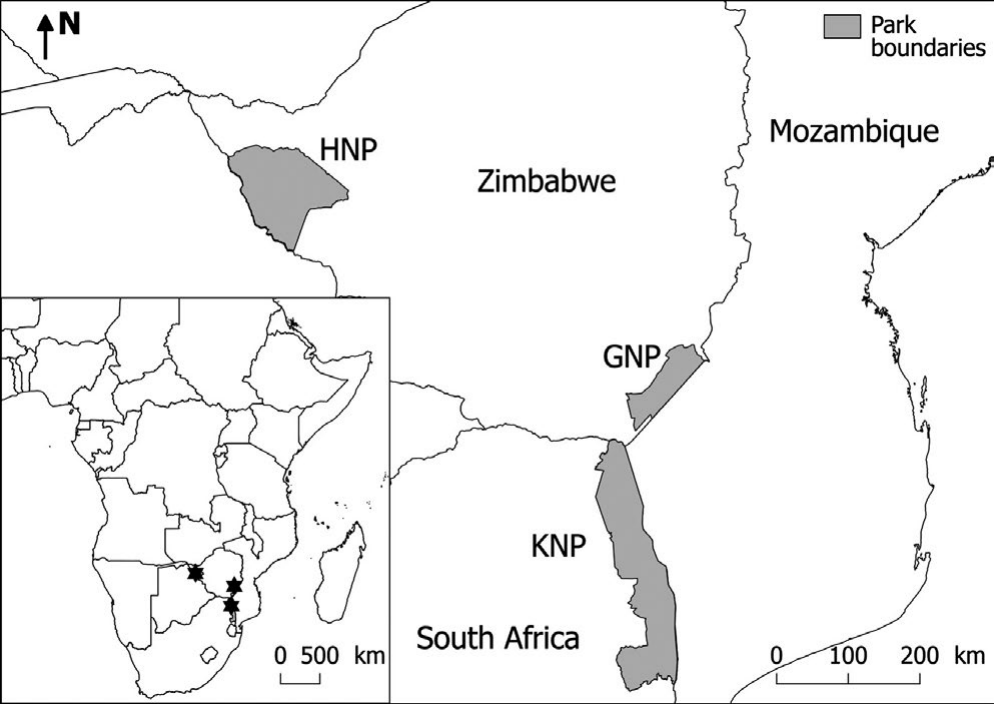
Location of the three study sites: Gonarezhou National Park (GNP) and Hwange National Park (HNP) in Zimbabwe, and Kruger National Park (KNP) in South Africa

The study was conducted across three sites: the eastern region of Hwange National Park (14,620 km^2^, HNP, Zimbabwe), the southern part of Gonarhezou National Park (5,053 km^2^, GNP, Zimbabwe), and in the north of Kruger National Park (18,989 km^2^, KNP, South Africa; Figure 2). Across the three study areas, the vegetation is a mosaic of bushland savanna, open grassland, and woodland (GNP: Gandiwa & Zisadza, 2010, HNP: Chamaillé‐Jammes, Fritz, & Murindagomo, 2006, KNP: Gertenbach, 1983). Annual rainfall across the three sites is similar with around 600 mm for HNP and 500 mm for GNP and KNP. The distribution of rainfall within the year is also similar between sites, with most rain falling between November and March (GNP: Gandiwa, Heitkönig, Eilers, & Prins, 2016, HNP: Chamaillé‐Jammes et al., 2006, KNP: Gertenbach, 1980). The core wet season was therefore defined as the period running from January 1st to March 31st (*n* = 90 days) and the core dry season from August 15th to October 31st (*n* = 78 days) for all sites. During the wet season, grass water content is high, and water is widely distributed in the landscape across numerous natural and artificial pans (HNP, KNP) or rivers (GNP, KNP). During the dry season, most natural pans dry up and water distribution in the range of Cape buffalo differs between sites. In GNP, water is only available in a few pools in the main river; in HNP, only artificial pumped waterholes provide water; in the north of KNP (Pafuri region), water is provided by a few permanent rivers and some pools which persist along the Limpopo river (GNP: Zvidzai, Murwira, Caron, & de Garine Wichatitsky, 2013, HNP: Chamaillé‐Jammes, Fritz, & Murindagomo, 2007, KNP: Purdon & van Aarde, 2017). The number and density of lions (*Pantera leo*), which is the most important predator of Cape buffalo, vary across the sites (Bauer & Van Der Merwe, 2004): In KNP, the population density of lions is between 5 and 8 lions/100 km^2^ (Ferreira & Funston, 2010), around 2–3 times higher than the density of lions in HNP (2.7 lions/100 km^2^, Loveridge, Searle, Murindagomo, & Macdonald, 2007), and up to 8 times higher than that in GNP (1–2.5 lions/100 km^2^, Bauer & Van Der Merwe, 2004, Groom, 2009).

### 2.2 Environmental covariates

We used one unpublished and two published vegetation maps, each covering a study area and using different vegetation classes, to create simpler and more homogeneous maps for our comparative analyses. We combined the vegetation classes of the original maps into three broad habitat types: (a) grassland, including areas dominated by grassland, or bushed grassland with sparse vegetation, (b) bushland, which consists of shrubby and bushy areas, and (c) woodland, encompassing deciduous, evergreen, or riverine forests (see Supporting Information S1 for detailed information on the classification process). In GNP and HNP, we identified the permanent waterholes following systematic monitoring of artificial and natural water pans within the home ranges of Cape buffalo groups studied here. This monitoring was implemented at the same periods as the deployment of the GPS collars (2011 in GNP and 2013–2014 in HNP). In KNP, the location of every permanent waterhole was recorded from Google Earth (Google Inc., Mountain View, CA) using photographic capture taken at different times of the year. Due to the presence of numerous natural pans at all sites, it was difficult to quantify water availability outside of the core dry season. Because of this and to avoid transitional periods, we restricted our analyses to the core of the wet and dry seasons (hereafter called wet and dry season, respectively). We considered water as a nonlimiting factor in the wet season (Bennitt, Bonyongo, & Harris, 2014; Cornélis et al., 2011).

### 2.3 Capture and collaring

Between 2008 and 2016, we tracked 47 adult female Cape buffalo across the three study areas (GNP: *n* = 12, HNP: *n* = 20, KNP: *n* = 15) using GPS collars. We focused on adult females, as adult males are known to leave subgroups and groups more often (Prins, 1996; Sinclair, 1977). All animals were captured by authorized personnel using established techniques (la Grange, 2006) and were observed returning to their subgroups after collaring operations. All field operations were conducted in accordance with the legal and permit requirements of the countries in which they were carried out.

The data acquisition periods extended from October 2008 to May 2011 in GNP, from April 2010 to January 2016 in HNP, and from June 2010 to July 2015 in KNP. Duration of the tracking varied between 19 and 1,013 days (median = 486) across individuals, and GPS loggers were scheduled to acquire locations at synchronous times (the top of the hour) every hour. We computed fix success rate within each season within each year for each individual, and we retained GPS data from 38 collared individuals for which the success rate was higher than 90%. Using this threshold, all selected individuals had at least one location per day within a single season (median = 24 locations/day). These individuals consisted of 10 groups: 2 in GNP, 4 in HNP, and 4 in KNP. The number of collared cows in each group varied between 1 and 7 (GNP groups, *n*_1_ = 4, *n*_2_ = 6; HNP groups, *n*_1_ = 7, *n*_2_ = 6, *n*_3_ = 1, *n*_4_ = 1; KNP groups, *n*_1_ = 5, *n*_2_ = 2, *n*_3_ = 5, *n*_4_ = 1). Of these, 31 females were tracked in both wet and dry seasons (GNP: *n* = 10, HNP: *n* = 11, KNP: *n* = 10) and 7 in only one season (HNP: *n* = 4, KNP: *n* = 3). This global dataset was used for a preliminary analysis (see section *Definition of association and fission–fusion events* below*)*, before some data selection processing for subsequent analyses (see *Statistical analyses* section).

### 2.4 Estimation of home ranges and home range overlaps

To estimate home range overlap (HRO) between individuals, we considered seasonal home ranges (HR) as the 90% utilization distribution during the dry and wet seasons for each year. Utilization distributions were computed using the movement‐based kernel density estimation method (MKDE, Benhamou & Cornélis, 2010) implemented in the “adehabitatHR” package (Calenge, 2007) for R v. 3.3.2 (R Development Core Team, 2016). Home range overlap between individuals was estimated using the Bhattacharyya's affinity index (Benhamou, Valeix, Chamaillé‐Jammes, Macdonald, & Loveridge, 2014). The index accounts for variation in the intensity of HR use and can take values from 0 (no overlap) to 1 (identical space use).

### 2.5 Definition of association and fission–fusion events

Fusion and fission events were defined as the point in space and time at which individuals merged and split up, respectively. Each fusion and fission event led to a period where individuals were in the same or in a different subgroup, here after, respectively, called “periods in the same subgroup” and “periods in a different subgroup.” To quantify individual association patterns and define fission and fusion events, we calculated the distance between synchronous locations for every pair of individuals (*i.e.,* dyad) that shared space (HRO > 0) for a given season (GNP: *n* = 104, HNP: *n* = 20, KNP: *n* = 47). Two buffalos were defined as being in the same subgroup if they were located simultaneously within a 1 km distance from each other. This distance threshold was determined using the group definition proposed by Cross, Lloyd‐Smith, and Getz (2005): A mixed‐sex group is a set of individuals that are within 1 km of one another. At this same distance, Polansky, Wittemyer, Cross, Tambling, and Getz (2010) showed that female Cape buffalo synchronized their movements, thus giving an estimate of the maximum diameter of a subgroup. As the activity synchronization between members of a subgroup/group is essential to ensure cohesion, the result of Polansky et al. (2010) confirms that beyond 1 km, subgroups are likely to split. Bennitt et al. (2018) identified fusion events when collared Cape buffalo were within 300 m of each other. Most recorded interindividual distances in our three sites occurred at short distances (0–300 m: 74.27% of all distances between dyads < 1 km apart, Supporting Information S2). This suggests that choosing a large threshold distance does not lead to consider as in the same subgroup individuals that would often be widely separated, but rather allows accounting for rare situations when individuals that are usually close by are further apart, for instance, when in opposite sides of a large traveling subgroup. The use of Bennitt et al.’s (2018) method to define the a priori distance threshold did not qualitatively affect the results of this study (Supporting Information S2). Additionally, the 1‐km distance threshold was more consistent with field observations where Cape buffalo subgroups may spread over such distances (>800 m) when traveling and arriving at a water point (pers. obs.). To minimize the number of false fission or fusion events resulting from infrequent erroneous locations, we also considered that two Cape buffalo were in the same subgroup when their distance was ≥1 km for ≤2 hr (time threshold, *t*_th,_). The influence of the chosen distance (*d*_th_) and time thresholds on further analyses was examined using a sensitivity analysis (Supporting Information S3). Lowering *t*_th_ would change the absolute number of fission and fusion events (Supporting Information S3) but is unlikely to alter the qualitative conclusions of our study. To calculate the proportion of time spent in the same subgroup, we created for each dyad a binary vector of association, with value “S” when individuals were in the same subgroup and “D” when they were in a different subgroup. When one value was missing between two association values (“S” or “D”) (i.e., the location of at least one of the two individuals had not been recorded), we substituted the missing value by the value of the previous hour (GNP: 0.44% of the data, HNP: 0.31% of the data, KNP: 0.97% of the data). From these association vectors, we derived (a) the proportion of time spent in the same subgroup, (b) the timing and location of fission and fusion events, and (c) the duration of each period that dyads spent in the same subgroup and in a different subgroup (number of consecutive hourly time steps in the same subgroup or in a different subgroup). We defined fusion events as the D*_t_*_‐1_ ‐> S*_t_* transition, from being in a different subgroup (D) at time *t‐1* to being in the same subgroup (S) at time *t*. Conversely, fissions are the opposite transition: S*_t_*_‐1_ ‐> D*_t_*. We excluded the periods containing at least one missing timestamp when calculating the duration of periods. The occurrence of fusion events was used to calculate the number of fusion events (by definition, the number of fission events is equal) per dyad per month as the total number of fusion events divided by the number of months of simultaneous tracking.

### 2.6 Statistical analyses

**Figure 3:**
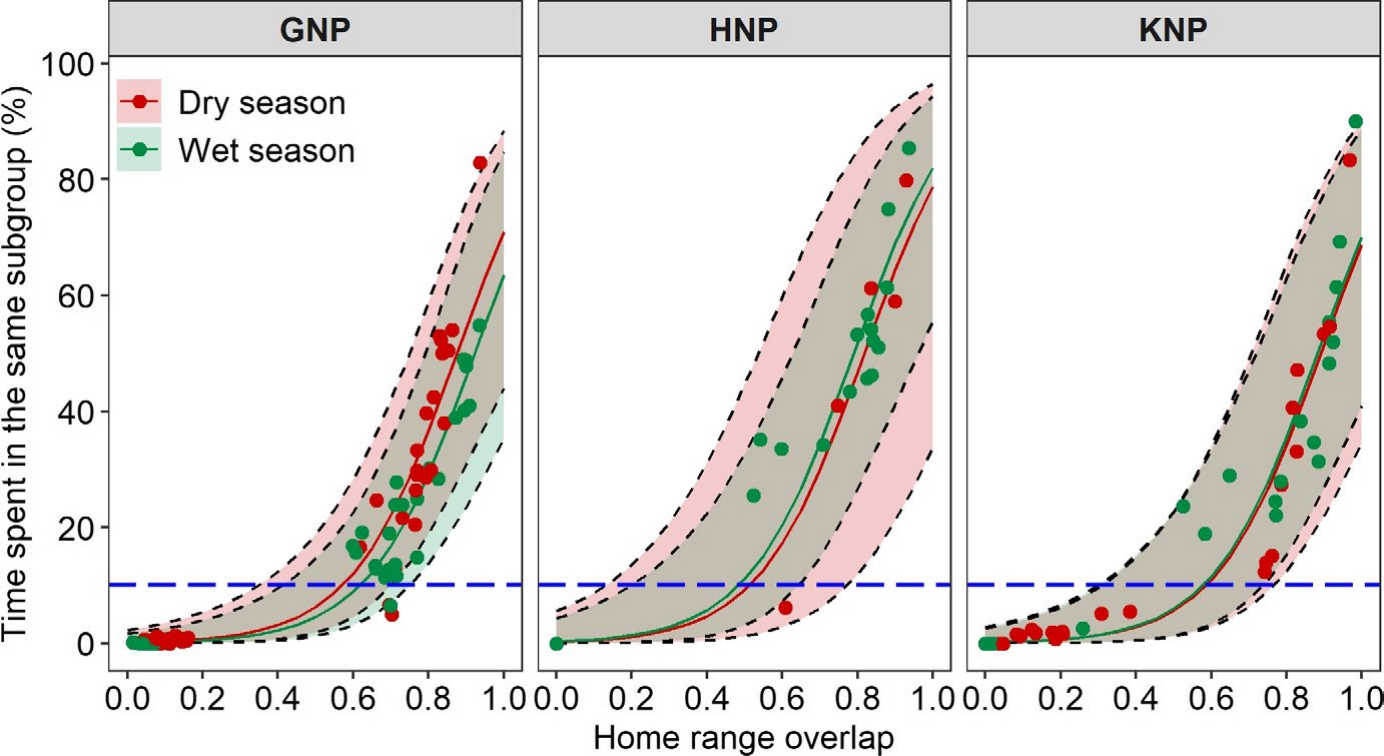
Relationship between the time spent in the same subgroup and HRO among pairs of Cape buffalo according to the study sites in dry (red) and wet (green) seasons. Points in corresponding colors are the observed values for each dyad per year and season. Solid lines represent the predictions from the model, and gray dashed lines represent 95% confidence intervals. Horizontal blue dashed line indicates the cut‐off value of 10% of time spent in the same subgroup

Animals from neighboring groups can occasionally be in the vicinity of one another by chance (e.g., by randomly using the same resource patches at the same time). To avoid qualifying these events as within‐group fission–fusion events, we restricted our analyses to dyads that spent a given amount of time in the same subgroup. To determine an appropriate cut‐off value, we investigated how the proportion of time that two individuals spent in the same subgroup was related to their HRO. We fitted a generalized (quasibinomial) additive mixed model with the proportion of time spent in the same subgroup as the response variable, and seasons (dry versus wet), sites (GNP, HNP versus KNP) and their interaction, and HRO as explanatory variables with dyad identity as a random effect. From this preliminary analysis, we restricted our subsequent analyses to dyads that spent ≥ 10% of their time in the same subgroup (i.e., corresponding to dyads within the same group, see Figure 3). In all subsequent analyses, we used the nested random effects of dyad identity within group identity to account for the repeated measures of dyads within the groups.

We investigated the stability of HRO and proportion of time spent in the same subgroup across seasons at the dyad level. For each dyad, we calculated the differences in HRO and proportion of time spent in the same subgroup between the dry season and the preceding wet season. To test whether these differences differed from 0 and varied between sites, we used two linear mixed models: The response variable was either (a) the seasonal difference in HRO or (b) the seasonal difference in time spent in the same subgroup and site was the unique explanatory variable in both models.

We then explored whether characteristics of the fission–fusion dynamics (i.e., the number of fusion events per month and the duration of periods that dyads spent in the same/different subgroup) varied across sites and seasons. Three generalized linear mixed models with negative binomial distributions of errors were fitted: The responses variables were either (a) the number of fusion events per month, (b) the duration of every period spent in the same subgroup, or (c) the duration of every period spent in a different subgroup. Sites, seasons, their interaction, and HRO were the explanatory variables. To analyze the distribution of fission and fusion events across the diel cycle, we ran two generalized additive mixed models with cubic splines and Poisson distribution of errors, with the number of (a) fission events or (b) fusion events per hour of the day per month as the response variables. The explanatory variables were site, season, and their interaction and the time of the day.

Finally, we explored whether fission and fusion events and periods spent in the same/in a different subgroup occurred in areas of the landscape differing in terms of distance to water (during the dry season) or vegetation type (during dry and wet seasons). The spatial location of fission and fusion event was defined as the average of the spatial coordinates of both individuals of the dyad. To describe habitat of each individual of a dyad when they were in a same or in a different subgroup, we grouped, for each individual, all locations when they were in a different subgroup and all locations when they were in the same subgroup. We calculated utilization distribution (90% UD using the MKDE approach) for those, resulting, for each individual of a dyad, in one UD when the individual was in the same subgroup than the other individual and one UD when the individual was in another subgroup. We generated 300 random points/km^2^ in each UD. The vegetation class and distance to the nearest water point were extracted at each fission and fusion location and at each random point in the UDs. To determine whether individuals of a dyad were more likely to fuse or merge, and be in the same subgroup or not, with changing distance to water and vegetation, we used generalized linear mixed models with a binomial distribution of errors. As distance to water was reliable and meaningful only during the dry season, we conducted a set of models for each season. In all cases, the response variable was whether the location was a fusion (scored 1) or fission (scored 0), and for a second set of models whether the location corresponded to a period where the individuals were in the same (scored 1) or in a different subgroup (scored 0). We used site, vegetation class, and their interaction as explanatory variables in the wet season models and site, vegetation class, distance to water, and their interaction in the dry season models.

For each analysis above‐mentioned, we used the Akaike information criterion corrected for small sample size (AICc) to test whether a simpler model, nested in the full model, would be more parsimonious (Burnham & Anderson, 2002). Model sets are presented in Table 1. We considered the most parsimonious model to be the model that had both a ∆AICc < 2 and the lowest number of explanatory variables (Arnold, 2010). The goodness‐of‐fit measure of the models was estimated by the adjusted R‐squared (Wood, 2017) for generalized additive models (Table 1—analyses 1, 7–8) and by the marginal pseudo‐R‐squared (Nakagawa, Johnson, & Schielzeth, 2017) for generalized linear mixed models (Table 1—analyses 2 to 6, 9–10) using the “performance” package (Lüdecke, Makowski, & Waggoner, 2019). Analyses were conducted using the “lme4” (Bates, Mächler, Bolker, & Walker, 2015), “mgcv” (Wood, 2011) and “glmmTMB” (Brooks et al., 2017) packages for R v. 3.3.2 (R Development Core Team, 2016).

**Table 1:** Summary of the candidate models fitted for each analysis. Response variables were modeled as a function of different combinations between HRO, site (GNP, HNP or KNP), season (dry or wet season), time of day, and event type (fission event, fusion event, when individuals are in the same subgroup but not at the moment of the fusion or when individuals are in different groups but not at the moment of the fission). The random effect was the dyad identity in the analysis 1, and the dyad identity nested within group identity in other analyses. For analyses 4–6, HRO was included in some models as an explanatory variable to control for the positive relationship between the number of fusion events or duration of periods in the same/different subgroup and HRO, as HRO positively affect the total time spent in the same subgroup (analysis 1)

Model	*df*	−2LL	∆AIC_c_	Adj. *R* ^2^/Pseudo‐$R_{\text{marginal}}^{2}$
*1. Relationship between proportion of time spent in the same subgroup and home range overlap*				
s(HRO) + site*season	**10**	**784**	**0.0**	**0.93**
s(HRO) + site	7	805	15.1	0.93
s(HRO) + site + season	8	803	15.1	0.93
s(HRO, by = site*season)	15	789	16.1	0.93
s(HRO)	5	833	38.9	0.90
s(HRO) + season	6	833	41.0	0.90
null	3	1,111	312.3	0.00
season	4	1,112	315.8	0.00
site	5	1,113	319.0	0.01
site + season	6	1,115	322.4	0.01
site*season	8	1,113	325.4	0.01
*2. Seasonal changes in home range overlap*				
**null**	**4**	**−67**	**0.0**	**0.00**
Site	6	−67	5.2	0.00
*3. Seasonal changes in proportion of time spent in the same subgroup*				
**null**	**4**	**368**	**0.0**	**0.00**
Site	6	367	4.3	0.08
*4. Number of fusion events*				
**HRO + season**	**6**	**440**	**0.0**	**0.39**
HRO + site +season	8	437	1.0	0.55
HRO + site*season	10	436	5.9	0.40
season	5	473	30.7	0.22
site + season	7	471	33.2	0.25
site*season	9	471	37.6	0.26
HRO	5	485	42.3	0.26
HRO + site	7	484	46.0	0.42
null	4	519	73.6	0.00
site	6	518	77.2	0.03
*5. Duration of periods in the same subgroup*				
**Site + season**	**7**	**15,761**	**0.0**	**0.12**
HRO + site +season	8	15,759	0.2	0.12
Site*season	9	15,761	3.6	0.11
HRO + Site*season	10	15,759	3.9	0.11
Season	5	15,770	4.9	0.04
HRO + season	6	15,768	5.3	0.04
Site	6	15,820	57.3	0.06
HRO + site	7	15,818	57.3	0.06
Null	4	15,828	61.3	0.00
HRO	5	15,826	61.3	0.00
*6. Duration of periods in a different subgroup*				
**HRO + site +season**	**8**	**16,293**	**0.0**	**0.22**
HRO + site*season	10	16,292	3.4	0.20
HRO + season	6	16,303	6.3	0.14
Site + season	7	16,355	60.5	0.17
Site*season	9	16,354	63.8	0.17
Season	5	16,362	63.8	0.06
HRO + site	7	16,365	70.5	0.17
HRO	5	16,376	77.5	0.09
Site	6	16,437	140.2	0.12
null	4	16,446	145.0	0.00
*7. Occurrence of fusion events during the diel cycle*				
**null**	**3**	**7,392**	**0.0**	**0.00**
Site	5	7,393	5.7	0.00
Season	4	7,441	51.1	0.03
Site + season	6	7,442	56.5	0.03
Site*season	8	7,444	62.1	0.03
s(Time of day)	4	7,473	82.9	0.09
s(Time of day) + site	6	7,475	89.0	0.09
s(Time of day) + season	5	7,508	119.9	0.12
s(Time of day) + site + season	7	7,510	126.6	0.12
s(Time of day) + site * season	9	7,512	131.9	0.12
*8. Occurrence of fission events during the diel cycle*				
**null**	**3**	**7,360**	**0.0**	**0.00**
Site	5	7,362	5.9	0.00
s(Time of day)	4	7,372	14.5	0.07
s(Time of day) + Site	6	7,375	20.9	0.07
season	4	7,409	51.2	0.03
Site + season	6	7,410	55.9	0.03
Site*season	8	7,409	59.2	0.03
s(Time of day) + season	5	7,426	70.0	0.10
s(Time of day) + site + season	7	7,428	75.8	0.11
s(Time of day) + site * season	9	7,428	80.1	0.11
*9. Probability of fusion (versus fission) in relation to vegetation class in wet season*				
**Vegetation**	**5**	**4,049**	**0.0**	**0.01**
Vegetation + site	7	4,047	1.4	0.01
Vegetation * site	11	4,045	8.1	0.01
null	3	4,073	19.7	0.00
Site	5	4,073	23.7	0.00
*10. Probability of being in the same subgroup (versus different subgroup) in relation to vegetation class in wet season*				
Vegetation * site	**11**	**2,588,930**	**0.0**	**0.03**
Vegetation	5	2,590,802	1861.8	0.00
Vegetation + site	7	2,590,800	1862.9	0.03
null	3	2,590,918	1972.0	0.00
Vegetation	5	2,590,914	1973.1	0.03
*11. Probability of fusion (versus fission) in relation to distance to water and vegetation class in dry season*				
Vegetation * distance to water * site	**20**	**1,158**	**0.0**	**0.13**
Vegetation * site + vegetation * distance to water + distance to water * site	16	1,171	4.6	0.10
vegetation * distance to water + distance to water * site	12	1,181	5.7	0.08
Vegetation + distance to water * site	10	1,186	7.3	0.07
Vegetation * site + distance to water * site	14	1,179	8.0	0.08
Vegetation * site + distance to water * vegetation	14	1,179	8.6	0.08
Vegetation * distance to water	8	1,192	8.8	0.06
Vegetation + distance to water	6	1,199	11.5	0.05
Vegetation * site + distance to water	12	1,187	11.9	0.07
Vegetation * distance to water + site	10	1,191	12.4	0.06
Vegetation	5	1,202	13.1	0.05
Vegetation * site	11	1,191	13.6	0.06
Vegetation + site +distance to water	8	1,198	14.9	0.05
Vegetation + site	7	1,202	17.1	0.05
Distance to water * site	8	1,207	24.1	0.05
Distance to water	4	1,223	32.0	0.02
Distance to water + site	6	1,222	35.3	0.02
null	3	1,235	42.0	0.00
Site	5	1,235	45.5	0.00
*12. Probability of being in the same subgroup (versus different subgroup) in relation to distance to water and vegetation class in dry season*				
Vegetation * distance to water * site	**20**	**1,473,506**	**0.0**	**0.03**
Vegetation * site + vegetation * distance to water + distance to water * site	16	1,473,681	166.4	0.03
vegetation * site + distance to water * site	14	1,473,867	348.6	0.03
Vegetation * site + vegetation * distance to water	14	1,474,048	530.4	0.03
Vegetation * distance to water + distance to water * site	12	1,474,263	740.4	0.03
Vegetation + distance to water * site	10	1,474,280	753.7	0.03
Vegetation * site + distance to water	12	1,474,307	784.5	0.03
Distance to water * site	8	1,474,416	886.2	0.03
Vegetation * distance to water	8	1,474,655	1,125.1	0.02
Vegetation * distance to water + site	10	1,474,655	1,128.7	0.03
Vegetation + distance to water	6	1,474,687	1,153.3	0.02
Vegetation + distance to water + site	8	1,474,687	1,156.8	0.03
Distance to water	4	1,474,866	1,328.4	0.02
Distance to water + site	6	1,474,866	1,331.9	0.02
Vegetation * site	11	1,486,983	13,458.9	0.01
Vegetation	5	1,487,329	13,792.4	0.00
Vegetation + site	7	1,487,327	13,794.7	0.01
null	3	1,488,183	14,642.8	0.00
Site	5	1,488,181	14,645.1	0.01

Notes:

For each model, the degree of freedom (*df*), deviance = −2*loglikelihood (−2LL), difference in AIC_c_ values between the best fit and model_i_ (∆AIC_c_), model fit estimated by the adjusted R‐squared (Wood, 2017) for GAMMs (analyses 1, 7–8 below), and the marginal pseudo‐R‐squared (Nakagawa et al., 2017) for GLMMs (analyses 2 to 6, 9–10 below)—Higher values indicate better model fit in both cases. The ranking was based on the ∆AIC_c_. The best model, *that is,* which had both a ∆AICc < 2 and the lowest number of explanatory variables, is shown in bold for each analysis. s(variable): explanatory variable with a spline effect.

## 3 RESULTS

### 3.1 Relationship between the proportion of time spent in the same subgroup and home range overlap

Home range overlap and the proportion of time that dyads spent in the same subgroup were positively and nonlinearly related (Figure 3). With a few exceptions, the very small proportion of time spent in the same subgroup (<10%) was associated with a small to moderate HRO (<0.4). Moderate time spent in the same subgroup (10% << 50%) could be associated with widely different HRO (0.5 << 0.9). Individuals spending more than 50% of their time in the same subgroup always had a very large HRO (>0.8). The most parsimonious model between the proportion of time spent in the same subgroup and HRO fit the data well and included the interaction effect between site and season (Table 1—analysis 1, Table S3—analysis 1).

All subsequent analyses were restricted to dyads that spent ≥ 10% of their time in the same subgroup in at least one season.

### 3.2 Seasonal stability of home range overlap and association patterns

**Figure 4:**
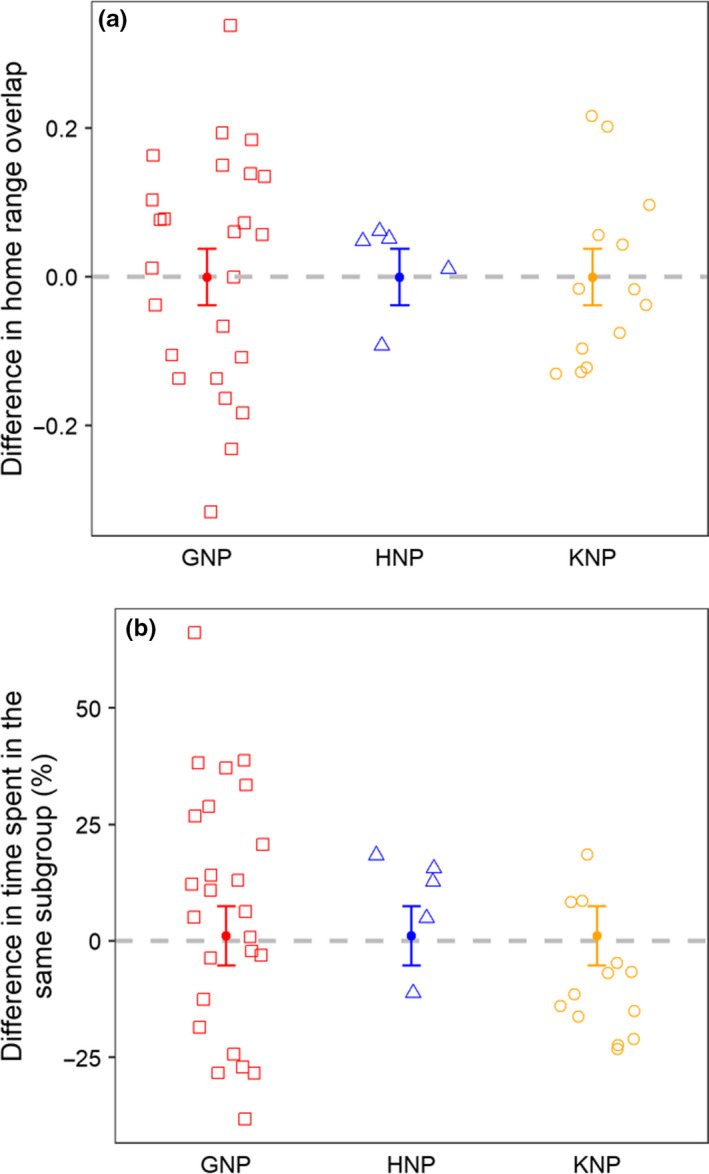
Influence of the study site on differences in (a) HRO between two individuals and (b) the proportion of time that two individuals spent in the same subgroup between the dry season and the preceding wet season. The open symbols correspond to observed data; the filled circles denote means, and the whiskers denote standard errors (SEs) for each site. Gray dashed line indicates no seasonal difference. A positive value means that two individuals spent more time in the same subgroup/had more HRO in the dry than in wet season

The most parsimonious models explaining the seasonal changes in both HRO and proportion of time spent in the same subgroup across all sites were the null models (Table 1—analyses 2–3). The average seasonal change (±SE) estimated for HRO was −0.001 ± 0.038 (95%CI: −0.075, 0.074) and 1.12 ± 6.37% (95%CI: −11.37, 13.60) for the proportion of time spent in the same subgroup (Figure 4, Table S3—analyses 2‐3).

### 3.3 Fission and fusion events

**Figure 5:**
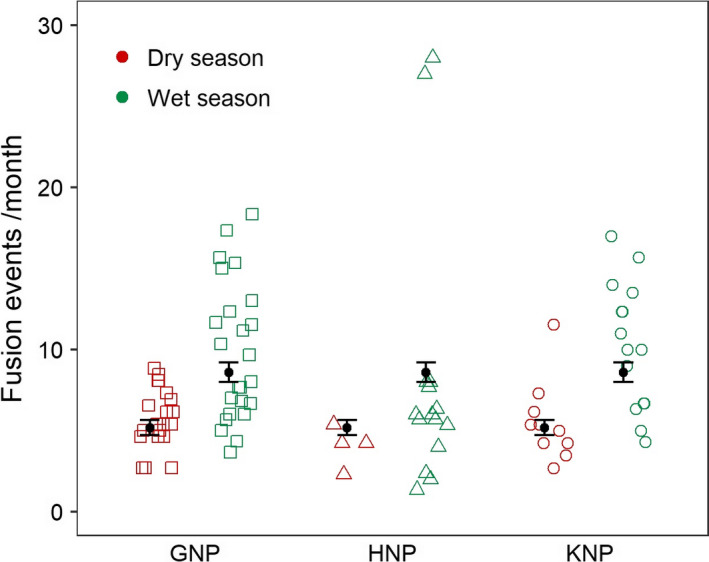
Effects of study site and season on number of fusion events per month per dyad. The open symbols give the observed values; the filled circles denote means, and the whiskers indicate SEs

Mean ± *SD* number of fusion events per month was 5.73 ± 1.86, 4.04 ± 1.28, and 5.54 ± 2.49 during the dry season in GNP, HNP, and KNP, respectively, and 9.83 ± 4.28, 8.22 ± 8.09, and 10.30 ± 3.92 during the wet season in GNP, HNP, and KNP, respectively. The most parsimonious model included the effect of HRO and season (Table 1—analysis 4), indicating that the frequency of fusion events was higher in the wet than in the dry season (Figure 5, Table S3—analysis 4).

**Figure 6:**
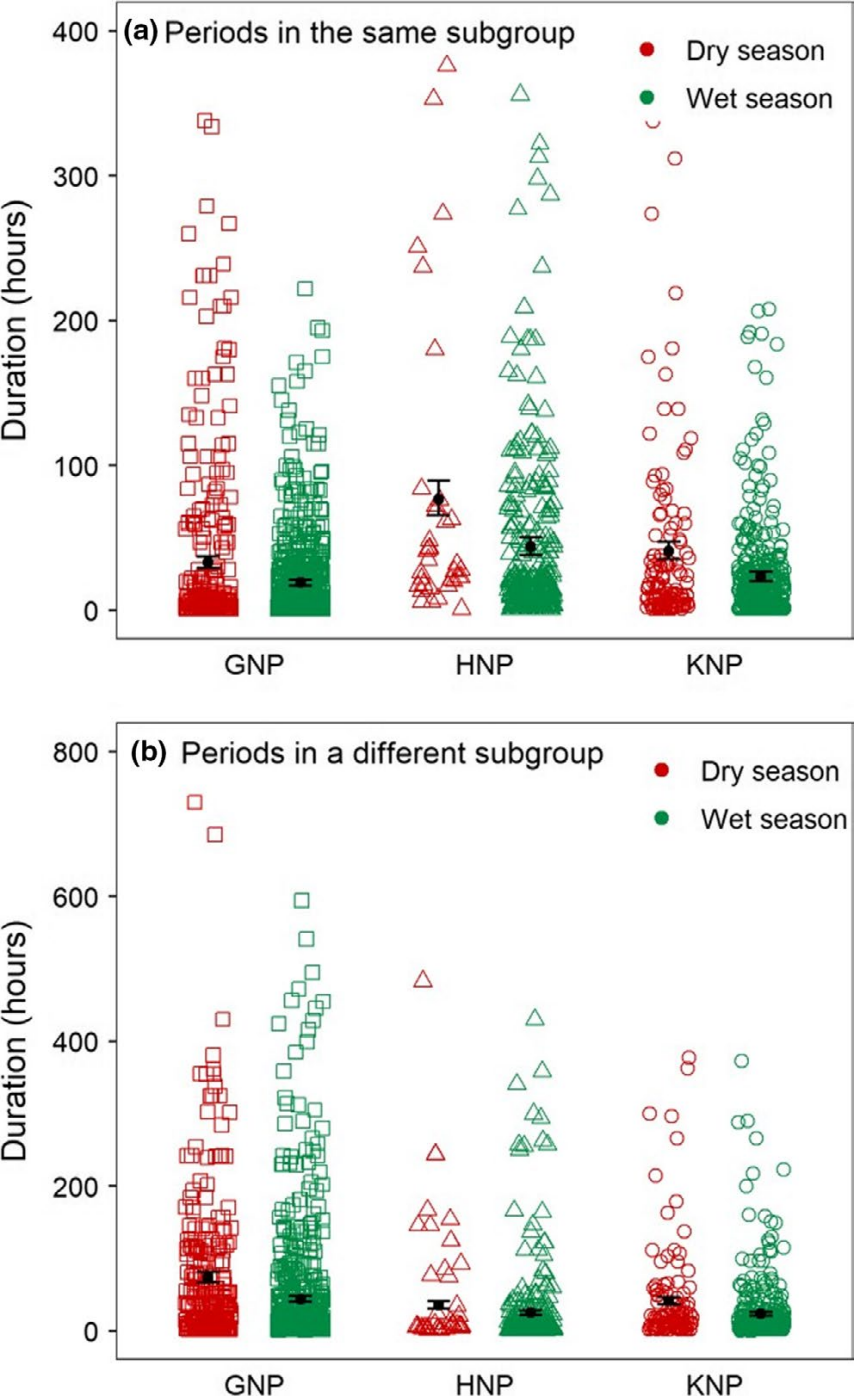
Effects of study site and season on duration of each (a) period spent in the same subgroup and (b) period spent in a different subgroup. The open symbols give the observed values; the filled circles denote means, and the whiskers indicate SEs

Mean ± *SD* duration of periods in the same subgroup was 35.6 ± 71.6, 88.4 ± 127, and 39.9 ± 65.2 hr during the dry season in GNP, HNP, and KNP, respectively, and 18.9 ± 29.6, 38.5 ± 70.1, and 23.6 ± 40.4 hr during the wet season in GNP, HNP, and KNP, respectively. Mean ± *SD* duration of periods in a different subgroup was 71.9 ± 118.0, 60.6 ± 97.9, and 47.0 ± 103.0 hr during the dry season in GNP, HNP, and KNP, respectively, and 42.7 ± 80.9, 20.9 ± 55.0, and 22.9 ± 42.7 hr during the wet season in GNP, HNP, and KNP, respectively. The most parsimonious model for the duration of periods in the same subgroup included effects of both season and site, and the most parsimonious model for the duration of periods in a different subgroup included the effect of HRO, season, and site (Table 1—analyses 5–6, Figure 6). For both duration of time spent in the same subgroup and duration of time spent in a different subgroup, the periods were shorter in the wet than in the dry season (Figure 6a‐b, Table S3—analyses 5–6). Irrespective of the season, the duration of the periods spent in the same subgroup (and in a different subgroup) were the shortest in GNP (HNP), slightly higher in KNP and much higher in HNP (GNP, Table S3—analyses 5–6). Overall, the effects of site and season explained only a small amount of the variability in the duration of both types of periods (pseudo‐*R* ^2^ in Table 1—analyses 5–6), suggesting that these variables may only slightly affect the duration of periods spent in a same or in different subgroups. Both types of period were on average short but very variable (Figure 6a‐b).

Both fission and fusion events occurred at any time of the day, but they occurred more frequently in the early morning (04h00–07h00) and from midafternoon to the early evening (15h00–19h00, not shown). However, the most parsimonious models on occurrence of fission and fusion events at diel cycle were the null models, suggesting that fission and fusion events occurred at any time of the day in all sites and in both seasons (Table 1—analyses 7–8, Table S3—analyses 7–8 for model results).

### 3.4 Environmental characteristics of fission and fusion events

**Figure 7:**
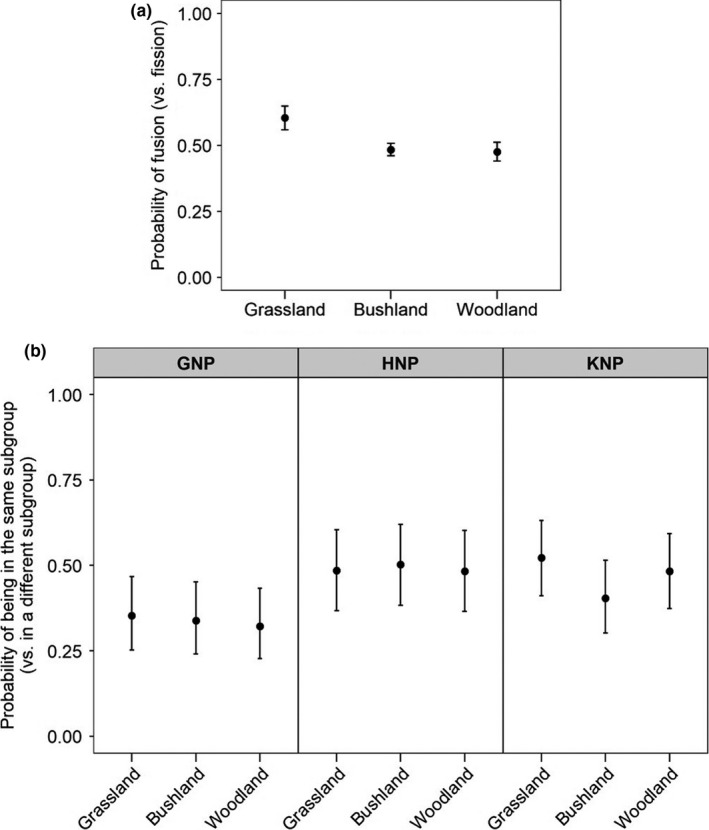
(a) Effect of vegetation in wet season on the probability of a fusion–fission event to be a fusion (*n*_grassland_ = 453, *n*_bushland_ = 1724, *n*_woodland_ = 761) and (b) effect of vegetation and site in wet season on the probability of being in the same subgroup versus being in a different subgroup (GNP – *n*_grassland_ = 46,285, *n*_bushland_ = 722,605, *n*_woodland_ = 100,632; HNP – *n*_grassland_ = 296,875, *n*_bushland_ = 304,701, *n*_woodland_ = 38,181; KNP – *n*_grassland_ = 39,936, *n*_bushland_ = 77,362, *n*_woodland_ = 55,368). Error bars represent 95% confidence intervals

During the wet season, the most parsimonious models explaining the probability that a fission–fusion event was a fusion only included the effect of the vegetation class (Table 1—analysis 9). Fusions were slightly more likely than fissions in grasslands, which was not the case in bushlands or woodlands (Figure 7a, Table S3—analysis 9). However, the model had a very low pseudo‐*R* ^2^ (Table 1—analysis 9). It should also be noted that most Cape buffalo GPS locations were in bushlands and most fusion events also occurred in bushlands (see Figure 7 caption). The most parsimonious model for the probability of being in the same subgroup versus in a different subgroup during the wet season included an interactive effect of vegetation class and site (Table 1—analysis 10). The pseudo‐*R* ^2^ of the model was very small (Table 1—analysis 10), and importantly here, within sites, vegetation had only minor, biologically irrelevant effects on the probability of being in the same subgroup (Figure 7b, Table S3—analysis 10).

**Figure 8:**
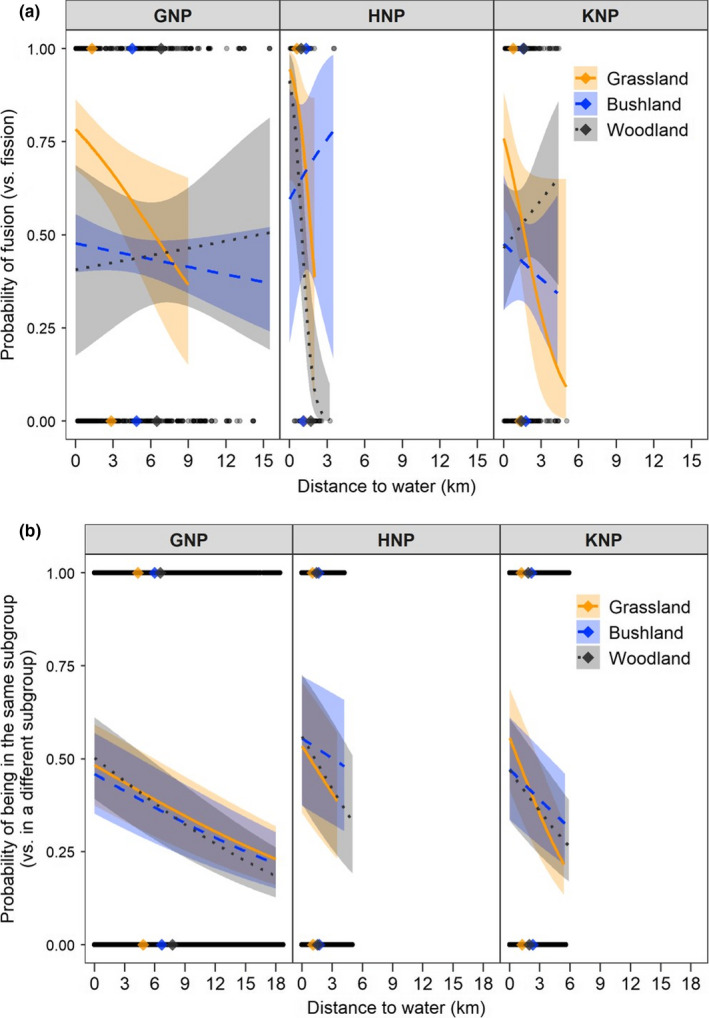
Effect of distance to water, vegetation, and site in dry season on the probability (a) of a fusion–fission event to be a fusion (GNP – *n*_grassland_ = 99, *n*_bushland_ = 430, *n*_woodland_ = 49; HNP – *n*_grassland_ = 21, *n*_bushland_ = 15, *n*_woodland_ = 46; KNP – *n*_grassland_ = 57, *n*_bushland_ = 78, *n*_woodland_ = 96) and (b) of being in the same subgroup versus being in a different subgroup (GNP – *n*_grassland_ = 106,797, *n*_bushland_ = 623,577, *n*_woodland_ = 106,638; HNP – *n*_grassland_ = 20,289, *n*_bushland_ = 45,521, *n*_woodland_ = 108,119; KNP – *n*_grassland_ = 27,111, *n*_bushland_ = 64,166, *n*_woodland_ = 47,786). Shaded areas represent 95% confidence intervals. Distance to water at the observed locations is shown using black dots. The colored dots show the mean distance to water for each vegetation class

During the dry season, the most parsimonious models explaining the probability of an event to be a fusion rather than fission and the probability of being in the same subgroup versus in a different subgroup included the interaction between vegetation class, distance to water and site (Table 1—analyses 11–12). At all sites, and in grasslands, fusions were progressively more likely than fissions as proximity to water increased (Figure 8a, Table S3—analysis 11). Similarly, two individuals were more likely to be in the same subgroup when they were closer to water, irrespective of the vegetation class (Figure 8b, Table S3—analysis 12). However, for both analyses, the pseudo‐*R* ^2^ values were low (Table 1—analyses 11–12), suggesting that a very large variability remained once the distance to water and vegetation class were accounted for. Additionally, Cape buffalo spent most of their time at distance to water at which the probability of fusion (versus fission) or the probability of being in the same subgroup (versus being in a different subgroup) was near 0.5, and the actual average distance to water did not differ much between fusion and fission events (Figure 8a), or between locations when Cape buffalo were in same of different subgroups in each vegetation class (Figure 8b). Finally, note that in GNP where Cape buffalo were often observed much farther away from water than in HNP and KNP, fusions commonly occurred away from water (Figure 8a).

## 4 DISCUSSION

As the factors hypothesized to drive fission–fusion dynamics may vary across populations, it is expected that the levels and patterns of fission–fusion dynamics may also vary. However, variability in fission–fusion dynamics across natural populations is currently known only from comparisons between independent studies that have used different approaches (Baden et al., 2016; Coles, Lee, & Talebi, 2012; Furuichi, 2009) or from experimental studies that investigate social dynamics in artificial settings (Kelley et al., 2011). Standardized comparative studies conducted *in natura* are essential for understanding how social, ecological, and demographic factors influence patterns of fission and fusion. Here, we address this important issue, by investigating the fission–fusion dynamics across three Cape buffalo populations living in similar environmental contexts. Patterns of fission and fusion were generally similar across all three populations suggesting that localized effects have little influence on adult female social dynamics in the species.

At all sites, the relationship between time spent in the same subgroup and the extent to which home range overlapped was positive. We found season and site effects on this relationship but, as these effects were small (see Figure 3) and only marginally improved model fit, we considered that the pattern was generally consistent across sites and seasons. However, the predictive power for a specific dyad might be low at all sites, as the proportion of time spent in the same subgroup remained highly variable for any given HRO, in particular when the overlap was large. Some of this unexplained variability might be linked to nonrandom associations that could not be controlled for when the GPS collars were deployed. While this study was conducted on adult females and therefore variability in association patterns is not linked to age/sex differences, female Cape buffalo may form preferred associations with close kin, as previously reported in species with fluid fission–fusion dynamics (elephants *Loxodonta africana,* Archie et al., 2006, eastern grey kangaroo *Macropus giganteus*, Best, Dwyer, Seddon, & Goldizen, 2014, bottlenose dolphins *Tursiops aduncus*, Frère et al., 2010). Body condition may also affect the association patterns, as Cape buffalo in Lake Manyara National Park (Tanzania) located in the rear of the herd, where conditions are worst, tended to split off more frequently from the herd (Prins, 1996). Possibly, variation in fission–fusion dynamics among our three studied populations could be due to differences in groups and/or subgroups size, but we were unable to record subgroup size over time using our methods.

This study revealed a highly dynamic fission–fusion system in all populations, as shown by the high number of fission and fusion events (on average, from 5.7 to 10.3 fusion events per month depending on sites and seasons, see Figure 5) and the short duration of periods during which two individuals were in the same or in a different subgroup (on average, between 18–88 hr and 20–71 hr, respectively, see Figure 6). Cape buffalo usually rests in the middle of the day and are most active during the early morning and afternoon (Cornélis et al., 2011; Megaze, Balakrishnan, & Belay, 2016; Valls‐Fox et al., 2018). During these periods, individuals are more likely to differ in their activities, with some individuals engaging in foraging activities, while others are moving to other food patches. Although inspection of the data suggested a slight trend for fission and fusion events to occur more during these periods (not shown), such diel patterns were not retained in the most parsimonious models, showing that there are no important cycles of fission–fusion dynamics throughout the day. It is thus not clear why dyads split, and the same question arises about why individuals fused regularly. This could be because forage is limited and heterogeneously distributed in semiarid areas such as our study sites. Cape buffalo could afford to congregate in areas where high‐quality resources are abundant, or conversely, be forced to come together when foraging patches are limited. The regular fusions could also be triggered by an intrinsic need to regroup (for instance, to obtain information, Fortin & Fortin, 2009).

The duration of periods spent in the same subgroup and in a different subgroup varied across sites, with the lowest durations observed in GNP, increasing in KNP and being the longest in HNP. The influence of the site was likely due to our large sample size, as the magnitude of this effect was small. These slight differences between sites could be real, but also be due to small interannual variations in fission–fusion dynamics between years, as not all sites were surveyed during similar period. The number of dyads tracked at each site during the common period (from June 2010 and May 2011) was too small, preventing us from testing such an effect. Overall, our results, therefore, point toward similar fission–fusion dynamics across all study populations, which differ strongly from the one observed in a population in the Okavango Delta (Botswana, Bennitt et al., 2018). The authors reported longer periods in the same subgroup than those observed in our study and lower fission and fusion rates: The mean duration of periods when individuals were in the same subgroup varied from 60 to 75 hr according to seasons (except in one rainy season where mean duration was 7.5 hr, our study—from 18 to 88 hr), and the mean number of fusion events per month varied between less than 1 and 3 (from 3 to 12 for the whole season based on dyads spent more than 10% of their total time in the same subgroup, our study—on average, between 5.7 to 10.3 fusion events per month). We consider unlikely that methodological differences in the definition of fission and fusion events (see Bennitt et al., 2018) could account for differences between this study and ours. This is particularly true as the authors used a distance threshold of 300 m, compared to 1,000 m in our study, to assume fusion. The use of a similar threshold between the two studies would have led to even larger differences. This comparison suggests a greater instability of subgroups in our populations and points toward resource conditions as being a driver of fission–fusion dynamics in Cape buffalo populations. GNP, HNP, and KNP are dominated by wooded semiarid savannas, whereas the area of the Okavango Delta where Bennitt et al. study took place is at the border of an alluvial plain where food quality and possibly water availability is greater (Bennitt et al., 2018). Future research should be conducted to further compare the environments of our sites and the Okavango Delta, such as the predation pressure, the size and distribution of food patches, the forage quality, and the access to water, which could be responsible for the variation in within‐group social dynamics observed between our sites and in the Okavango Delta.

The observed seasonal differences in the frequency of fusion events and the duration of both types of period hint at the role of resource condition as a driver of fission–fusion dynamics. At our study sites, despite the absence of seasonal changes in HRO or the proportion of time spent in the same subgroup, Cape buffalo usually split and associated more frequently and were in a different or in the same subgroup for shorter periods during the wet season when resource availability was high. As large Cape buffalo groups split more frequently than smaller ones (Prins, 1989), one could hypothesize that in the wet season, individuals occur in larger, more fluid subgroups than in the dry season when they would occur in smaller, more stable subgroups. An increase in subgroup size during the wet season has been shown in other species with fission–fusion dynamics (spider monkeys *Ateles belzebuth belzebuth*, Shimooka, 2003, blackbuck *Antilope cervicapra*, Isvaran, 2007, Thornicroft's giraffe *Giraffa camelopardalis*, Bercovitch & Berry, 2010) and in Cape buffalo (Sinclair, 1977). This would suggest that Cape buffalo has evolved a strategy to limit intragroup competition for food during the dry season while trying to benefit from larger aggregations (*e.g.,* protection against predators for newborns), at least temporarily, in the wet season when food competition is reduced. Additionally, Cape buffalo is in much poorer body condition during the dry season than the wet season (Beechler, Jolles, & Ezenwa, 2009), possibly reflecting an increased susceptibility to diseases during this period (Caron, Cross, & Du Toit, 2003; Ezenwa & Jolles, 2011). Being in smaller groups in dry season would help them to reduce pathogen transmission among individuals. During the wet season, the cost of social cohesion is expected to be lower, yet we found that individuals were in the same subgroup for shorter amounts of time. Why they stay in the same subgroup for shorter durations during the wet season remains unexplained but could be linked to resource availability. As resources are highly available, Cape buffalo may prefer to split more often and stay in the same subgroup for a shorter time to exploit available habitat more efficiently. Conversely, the low resource availability in the dry season could force Cape buffalo to congregate in the few patches where resources are plentiful and stay longer in these areas.

Much of the research on fission–fusion dynamics published to date has relied on direct observations and the recording of temporal changes in subgroup size, but these are often conducted on a small number of groups (Baden et al., 2016; Lehmann & Boesch, 2004; Pinacho‐Guendulain & Ramos‐Fernández, 2017). The lack of data on subgroup size is a limitation in this study, but using GPS tracking technology offers an individual viewpoint by describing fission–fusion dynamics of dyads in various groups at several sites using a unified analysis. However, it is worth noting that the constraints associated with this technology (e.g., deployment costs) have limited the number of individuals to be monitored simultaneously within the same group. Fission and fusion events involving noncollared animals have not been recorded, but it is unlikely that the behavior of those animals was highly different from our collared Cape buffalo and that the biases related to sample size were heterogeneous across sites and seasons. Consequently, the differences observed across sites and seasons should remain valid. Despite limitations, GPS tracking provides new information about whether local environmental conditions affect where fission and fusion events occur. In particular, our GPS data allowed us to test the predictions that Cape buffalo subgroups would be more likely to meet, that is, to experience a fusion event, and to remain together (a) in open habitats, (b) near water sources. The data provided mixed support for these predictions. Our wet season data suggested that grasslands are more likely to be associated with fusion than fission events. However, this effect was small, likely not biologically relevant, and subgroups were not more likely to be found with other subgroups than when in other vegetation class. Therefore, by itself, vegetation openness does not appear to be a determinant of fission–fusion dynamics in Cape buffalo populations. During the dry season, grasslands in areas located near water did appear to be more associated with fusion than fission events, which was generally not the case of other vegetation types. We assume here that, when Cape buffalo subgroups come to drink, vegetation openness might facilitate fusion by facilitating detection of other subgroups that are also coming to drink or have just been drinking. It is also possible that subgroups stay longer in grassland patches, foraging, thus giving more time for other subgroups to arrive and merge. The near presence of water appears crucial, however, for this to occur. As Cape buffalo subgroups were also more likely to be found in the same subgroup when closer to the water, irrespectively of the vegetation type they were in, it overall appears clear that the need for Cape buffalo to drink often (e.g., Valls‐Fox et al., 2018) associated with a limited number of water sources increases the likelihood of fusion during the dry season. Nevertheless, it is important to note that vegetation openness and importantly, distance to water are only weak predictors of whether fusion or fission will occur, or whether individuals will be observed in the same subgroup. Models including these environmental variables explained very little of the observed variability in where fusion and fission events occurred, or in the probability of observing subgroups together. In general, the average distance to water of fusion and fission events was similar, when compared to the whole range of distance to water experienced by Cape buffalo. This was also true for the average distance to water when individuals were in the same subgroup or not. Thus, our results agree with findings in Cape buffalo population from the Okavango Delta, where the average distance to permanent water between dry season fission and fusion events did not differ (Bennitt et al., 2018). We generally conclude that, although water distribution affects Cape buffalo space use during the dry season, it has, unexpectedly, only a minor impact on the spatial dynamics of fission–fusion events. However, GPS data should now be combined with behavioral observations to better understand the role of water availability as well as the ultimate causes (*e.g.,* social decisions) of fission–fusion dynamics.

## 5 CONCLUSIONS

Our study provides the most comprehensive description of the dynamics of association patterns in Cape buffalo reported so far. Cape buffalo in Hwange, Gonarezhou, and Kruger National Parks form associations based on a shared home range but loose temporal associations. These associations occur for generally short periods of time, and levels of fission–fusion dynamics are generally consistent across populations, with no strong environmental determination of where and when fusion and fission events occur. Strikingly, we found variability in fission–fusion dynamics across dyads within the same population, suggesting that further studies should now focus on identifying the factors underlying this heterogeneity. Such studies will be critical for (a) gaining a better understanding of drivers of fission–fusion dynamics across species (Sueur et al., 2011) and (b) improving our ability to understand and predict the consequences of social dynamics on other biological processes, such as the transmission of important pathogens (e.g., tuberculosis) that is a key concern in Cape buffalo populations (de Garine‐Wichatitsky et al., 2010).

## CONFLICT OF INTEREST

None declared.

## AUTHOR CONTRIBUTION

**Elodie Wielgus:** Conceptualization (equal); Data curation (equal); Formal analysis (lead); Methodology (equal); Writing‐original draft (lead); Writing‐review & editing (lead). **Daniel Cornélis:** Conceptualization (equal); Data curation (supporting); Funding acquisition (lead); Methodology (supporting); Resources (equal); Writing‐review & editing (equal). **Michel de Garine‐Wichatitsky:** Data curation (supporting); Resources (equal); Writing‐review & editing (supporting). **Bradley Cain:** Formal analysis (supporting); Funding acquisition (lead); Writing‐review & editing (equal). **Hervé Fritz:** Resources (equal); Writing‐review & editing (supporting). **Eve Miguel:** Data curation (supporting); Writing‐review & editing (supporting). **Hugo Valls‐Fox:** Data curation (supporting); Writing‐review & editing (supporting). **Alexandre Caron:** Conceptualization (equal); Data curation (equal); Methodology (equal); Resources (equal); Writing‐review & editing (equal). **Simon Chamaillé‐Jammes:** Conceptualization (equal); Data curation (equal); Formal analysis (lead); Methodology (equal); Resources (equal); Writing‐original draft (lead); Writing‐review & editing (lead).

## Supporting information

supinfo

## Data Availability

Data are publicly available from Dryad digital repository: https://doi.org/10.5061/dryad.1c59zw3sn.
