# Supplementary material for: Are fission–fusion dynamics consistent among populations? A large‐scale study with Cape buffalo
Source: Ecol Evol. 2020 Aug 11;10(17):9240–56. doi: 10.1002/ece3.6608 (PMC7487245; doi:10.1002/ece3.6608)
Supplement: Supplementary file 1 — supinfo [file ECE3-10-9240-s001.docx]

**SUPPORTING INFORMATION**

1. **Building a simplified habitat map**

We used vegetation maps that were available to us: an unpublished map for GNP (provided by D. Cornélis, available upon request) and published maps for the two other sites: Arrault et al (2018) for HNP and Pretorius and Pretorius (2015) for KNP. For GNP, the five classes of vegetation that were initially described, based on the vegetation structure, were treated as follows in our simplified maps: the class ‘open bushland’ was treated as ‘Grassland’ (there was no ‘grassland’ class in the original map); ‘dense bushland’ and ‘wooded shrubland’ as ‘Bushland’; and ‘riverine forest’ and ‘woodland’ as ‘Woodland’. For KNP, eight classes of natural vegetation were initially considered (see Pretorius and Pretorius 2015). We treated ‘grassland’ and ‘sparse vegetation’ classes as ‘Grassland’; ‘bushland’ and ‘open bushland’ as ‘Bushland’; and ‘woodland’, ‘riverine forest’, ’open woodland’ and ‘open riverine forest’ as ‘Woodland’. In GNP and KNP, the three types of non-vegetated or non-natural vegetation land cover classes (bare soil, cultivated areas and water) were aggregated into a ‘null’ class. For HNP, there were seven habitat types in the original habitat map: ‘grassland’ and ‘bushed grassland’ were treated as ‘Grassland’; ‘bushland’ and ‘scrubland’ as ‘Bushland’; and ‘deciduous woodland’ (2 classes) and ‘evergreen woodland’ as ‘Woodland’.

*References*

Arraut EM, Loveridge AJ, Chamaillé-Jammes S, Valls-Fox H, Macdonald DW. 2018. The 2013–2014 vegetation structure map of Hwange National Park, Zimbabwe, produced using free satellite images and software. Koedoe. 60:1–10.

Pretorius E, Pretorius R. Improving the potential of pixel-based supervised classification in the absence of quality ground truth data. 2015. South African J Geomatics. 4:250.

**2. Comparison of the fission-fusion parameters estimated using our method and the method described by Bennitt and colleagues (2018) to define the association between two individuals**


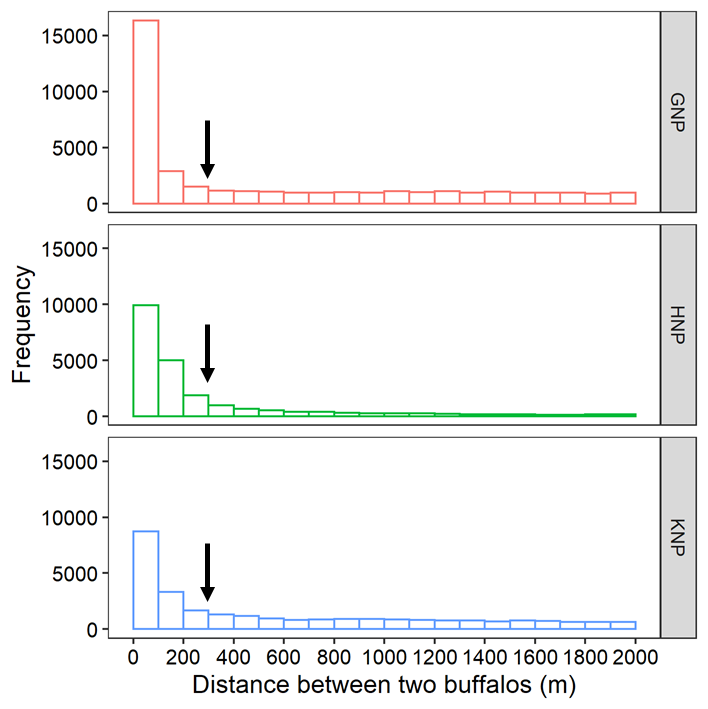
In order to explore the influence of how we define the subgroup membership on the fission-fusion dynamics, we compared the values of main results from our paper with those estimated if we had used the methodology of Bennitt et al (2018) to define associations. To identify associations and fusion events, the method used by Bennitt et al (2018) consisted in generating a histogram of the frequency of dyad Euclidian distances and using the distance threshold (d_th_) as when there was a notable drop in the frequency of observations. According to this method, we also define associations as when individuals were separated by < 300 m (Fig. S1) and we applied the same fluidity as Bennitt et al (2018, i.e. individuals separated by > 300 m for less than 3 hours were considered in the same subgroup).

**FIGURE S1** Histogram showing frequency of occurrence of Euclidean distance < 2000 m between Cape buffalo dyads in Gonarezhou (GNP) and Hwange National Parks (HNP), Zimbabwe and Kruger National Park (KNP), South Africa. The arrows mark the threshold distance for identifying fusion between dyads.

Here, we show the values for the main results of our article (i.e. the proportion of total time spent in the same subgroup, the number of fusion events /month and the duration of the periods spent in the same or a different subgroup) using the methodology described by Bennitt et al (2018), in comparison with the values estimated using our method. In our paper, we described the dynamics of fission and fusion events for the dyads spending at least 10% of their total time in the same subgroup (in order to avoid inclusion of intergroup contacts), we thus calculated the number of fusion events/month and the duration of the periods using the Bennitt et al’s method for the same dyads (even if they can spend less than 10% of their total time in the same subgroup when we use d_th_ = 300 m). The results obtained with the Bennitt et al’s method are graphically represented in the Figures S2-S4 and are compared with those obtained in our paper in Table S1-S2. As expected, changing the distance threshold qualitatively alters the results: in general, decreasing d_th_ leads to a decrease in the duration of periods spent in the same subgroup. However, despite the large difference in the chosen d_th_ (our d_th_ is three times higher than the one of Bennitt et al.), the values estimated with the two methods are generally of the same order of magnitude. Additionally, the most parsimonious models based on the AICc on the results obtained with the Bennitt et al’s method were the same as the most parsimonious models in our paper (results not shown), indicating that the choice of d_th_ should not qualitatively alter the conclusions of our work.

**TABLE S1** Mean ± SD number of fusion events per month estimated with the method used by Bennitt et al (2018) and with our method.

| Site | Season | Mean ± SD | |
| --- | --- | --- | --- |
|  |  | Bennitt et al’s method (d_th_ = 300 m) | Our method (d_th_ = 1 km) |
| GNP | Dry | 4.17 ± 1.45 | 5.73 ± 1.86 |
| GNP | Wet | 7.70 ± 4.28 | 9.83 ± 4.28 |
| HNP | Dry | 5.10 ± 1.45 | 4.04 ± 1.28 |
| HNP | Wet | 12.5 ± 10.8 | 8.22 ± 8.09 |
| KNP | Dry | 6.15 ± 2.34 | 5.54 ± 2.49 |
| KNP | Wet | 12.2 ± 4.57 | 10.3 ± 3.92 |

**TABLE S2** Mean ± SD duration of periods when individuals are in the same or in different subgroups estimated with the method used by Bennitt et al (2018) and with our method.

| Site | Season | Mean ± SD | |
| --- | --- | --- | --- |
|  |  | Bennitt et al’s method (dth = 300 m) | Our method (dth = 1 km) |
| In the same subgroup | | | |
| GNP | Dry | 47.0 ± 61.5 | 35.6 ± 71.6 |
| GNP | Wet | 16.2 ± 22.4 | 18.9 ± 29.6 |
| HNP | Dry | 68.1 ± 80.5 | 88.4 ± 127.0 |
| HNP | Wet | 22.4 ± 38.0 | 38.5 ± 70.1 |
| KNP | Dry | 30.7 ± 43.5 | 39.9 ± 65.2 |
| KNP | Wet | 14.0 ± 20.8 | 23.6 ± 40.4 |
| In different subgroups | | | |
| GNP | Dry | 97.2 ± 209.0 | 71.9 ± 118.0 |
| GNP | Wet | 51.9 ± 106.0 | 42.7 ± 80.9 |
| HNP | Dry | 51.9 ± 105.0 | 60.6 ± 97.9 |
| HNP | Wet | 17.2 ± 44.7 | 20.9 ± 55.0 |
| KNP | Dry | 41.8 ± 74.2 | 47.0 ± 103.0 |
| KNP | Wet | 24.2 ± 46.4 | 22.9 ± 42.7 |


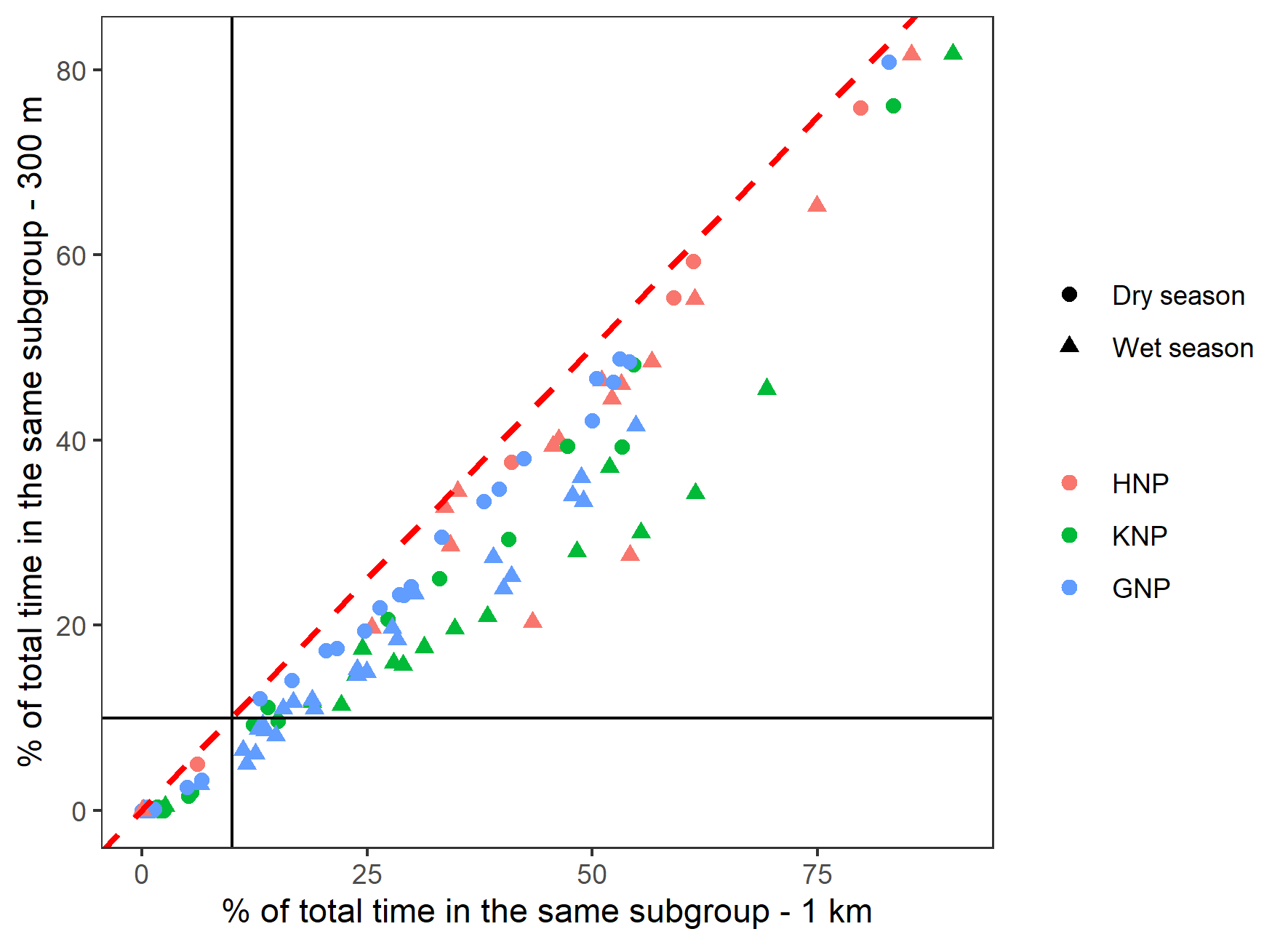


**FIGURE S2** Correlation between the proportion of total time spent in the same subgroup estimated using the method of Bennitt et al (2018, d_th_ = 300 m) and the proportion of total time spent in the same subgroup estimated with our method (d_th_ = 1 km).


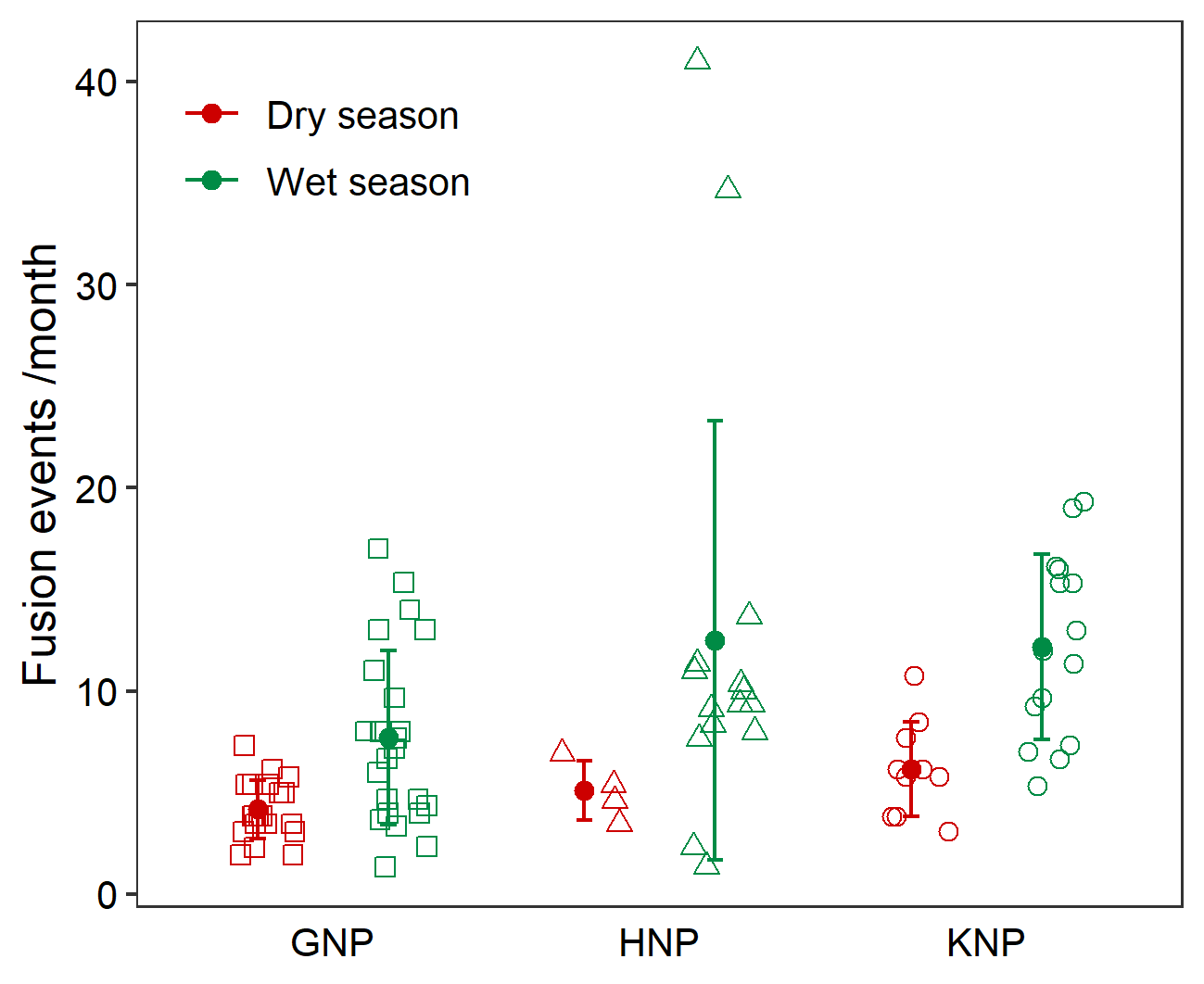


**FIGURE S3** Effects of study site and season on the number of fusion events per month per dyad, calculated with the Bennitt et al's method. The open symbols give the observed values; the filled circles denote means and the whiskers indicate SDs.


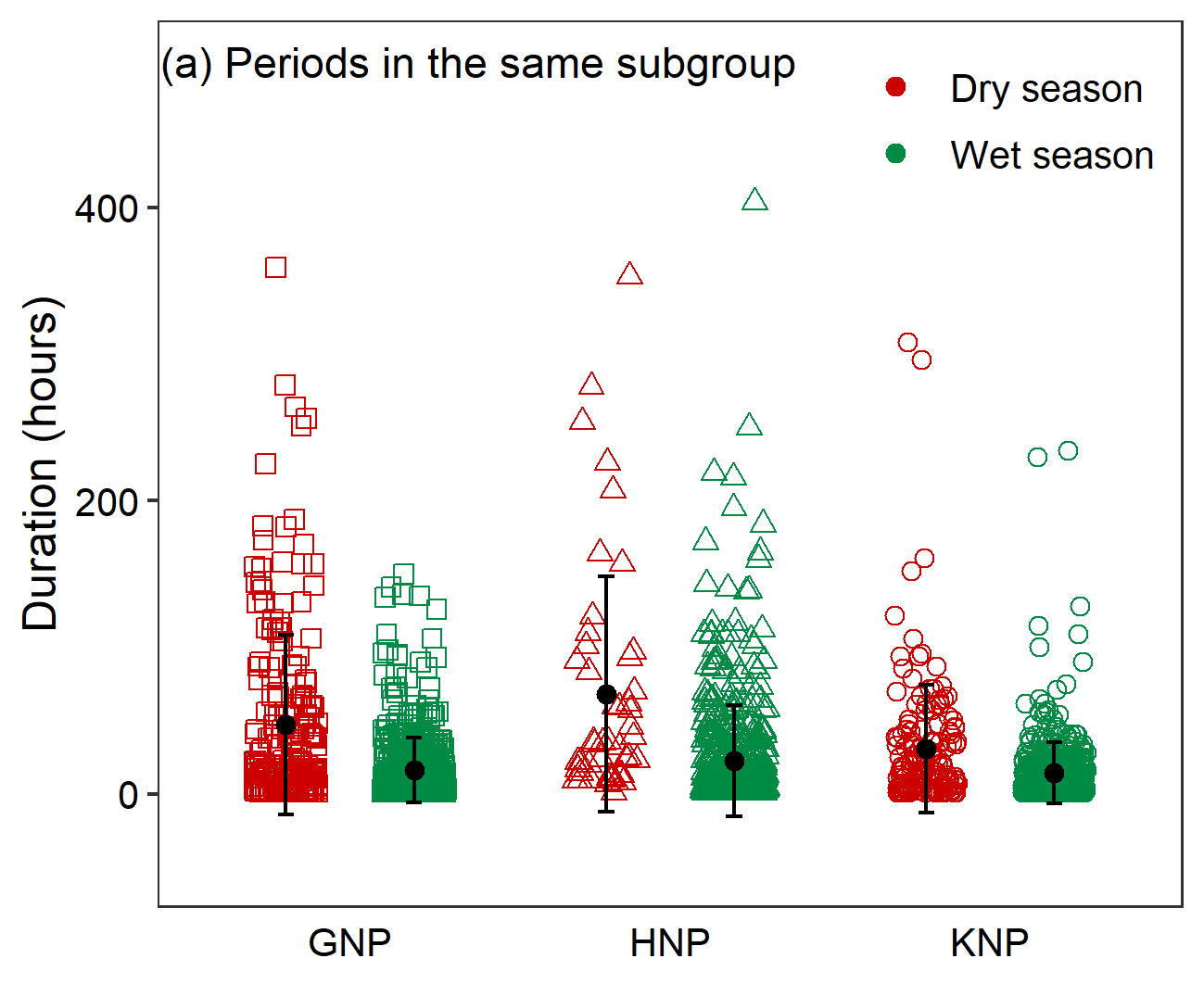


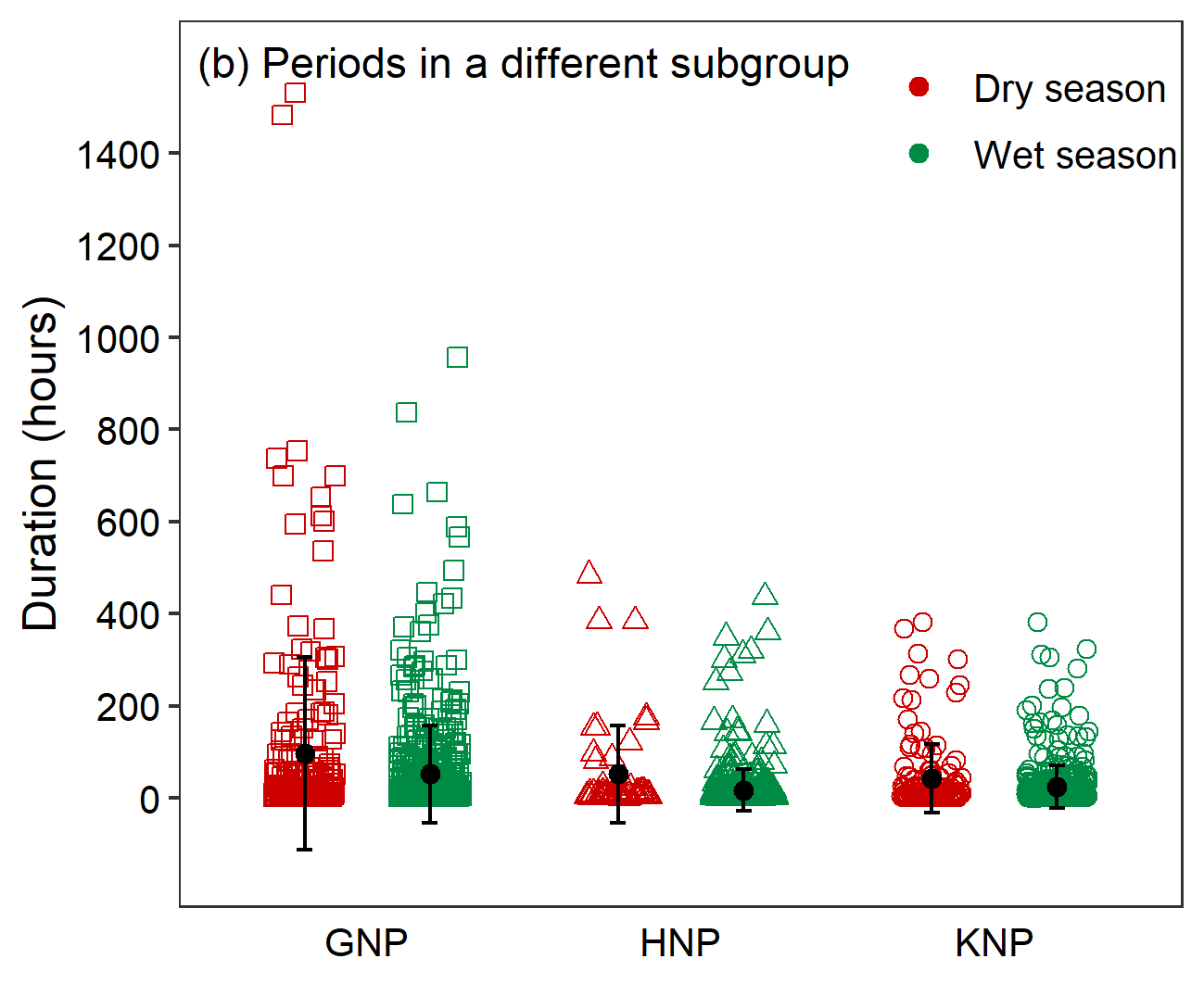


**FIGURE S4** Effects of study site and season on duration of each (a) period spent in the same subgroup and (b) period spent in a different subgroup estimated with the Bennitt et al’s method. The open symbols give the observed values; the filled circles denote means and the whiskers indicate SDs.

1. **Sensitivity analysis**

In order to quantify association patterns and define fission and fusion events, we needed to define under which conditions two individuals were considered in the same subgroup. For this purpose, we used a distance threshold d_th_ = 1000m (Cross et al. 2005, Polansky et al. 2010). When the distance between synchronous locations of two individuals was ≤ d_th_, individuals were considered as being in the same subgroup. The use of a distance threshold could easily lead to spurious breaks in association patterns when distances beyond the threshold are recorded for a short period. This can occur either because the distance between the individuals is close to the threshold and sometimes just above for a short, non-biologically meaningful duration, or because the GPS location for at least one of the individuals has a significant error (95% of GPS fixes were within 25m of the true position, pers. obs.). Such conditions are unlikely to be biologically meaningful in the context of our study, and we therefore also considered a time threshold t_th_, considering that two individuals were still in the same subgroup if the inter-individual distance was ≥ d_th_ for a duration ≤ t_th_.

To choose the most appropriate values for t_th_ and explore the effect of a d_th_ = 1000m on our further analyses, we evaluated how robust our estimations of (i) the proportion of total time that two individuals spent in the same subgroup and (ii) the number of fusion events per month, were to changes in d_th_ and t_th_ values. We did this at each study site and investigated d_th_ values ranging from 100 m to 3100 m and t_th_ values ranging from 2 h to 6 h. To calculate (i) the proportion of total time that two individuals spent in the same subgroup and (ii) the number of fusion events per month, we created for each dyad and each combination of values of d_th_ and t_th_, a binary vector of association, with value ‘S’ when individuals were in the same subgroup and ‘D’ when they were not. When one value was missing between two values (‘S’ or ‘D’) (i.e. the location of at least one of the two individuals had not been recorded), we substituted the missing value by the value of the previous hour. We defined fusion events as the D_t-1_ -> S_t_ transition, from being in a different subgroup (D) at time *t-1* to being in the same subgroup (S) at time *t*. Conversely, fissions are the opposite transition: S_t-1_ -> D_t_. From these association vectors, we derived (1) the proportion of time spent in the same subgroup (throughout simultaneous tracking) and (2) the number of fusion events per month, calculated as the total number of fusion events divided by the number of month of simultaneous tracking.

We explored the relationships between the proportion of time spent in the same subgroup or the number of fusion events per month and the values of d_th_, for each value of t_th_. We did this by fitting generalized additive mixed models (GAMM) with the proportion of time spent in the same subgroup or the number of fusion events per month as the response variable, d_th_ as explanatory variable and dyads as random effects. Models were fitted using the “mgcv” package in R (Wood 2011).

The proportion of time spent in the same subgroup and the number of fusion events per month were not very sensitive to the value of t_th_ in the three sites, except in HNP for low values of d_th_ where the number of fusion events per month varied greatly with t_th_ (Figs. S5b & S5e).

The proportion of time spent in the same subgroup was most sensitive to d_th_, resulting in greater time spent together as d_th_ increased in all sites (Fig. S5a-c). This was expected because, as d_th_ increases, extra locations are accounted for as being time spent together. The relationship between the number of fusion events and d_th_ is more complex and differ between sites. In HNP and to a lesser extent in KNP, increasing dth decreased the number of fusion events observed (Fig. S5e-f). This was expected because, as d_th_ was increased, buffalos were considered in the same subgroup for longer periods, leading to a reduced number of fusion events. Differences in the shape of the relationship between HNP and KNP suggest that dispersion of individuals within the herds differ, with animals being more dispersed in HNP. Unexpectedly, in GNP, increasing d_th_ increased the number of fusion events detected (Fig. S5d). We explain this observation by the regular proximity of the two groups studied, which share space significantly. As d_th_ increases, it is likely that times when herds were in close proximity became counted as a fusion event.

In the light of these results, a d_th_ value of 1000m for the analyses seemed to be able to account for the effect of small-scale dispersion of individuals in HNP (which was not of interest here). The choice d_th_ did not influence the number of fusion events calculated in KNP, and it would affect the results for GNP only moderately, in a way that could be understood thanks to this sensitivity analysis. As, at this d_th_ value, the proportion of time spent in the same subgroup and the number of fusion events were little sensitive to the value of t_th_ in the three sites, we selected the minimal t_th_ value, i.e. 2h, for the analyses presented in the main text. Note that overall, this sensitivity analysis showed that moderate changes in the values used for defining these thresholds would not alter our results qualitatively.

**FIGURE S5** Results of the sensitivity analyses for each site (GNP: Gonarezhou National Park; HNP: Hwange National Park; KNP: Kruger National Park). (a), (b), (c): Changes in the proportion of time that two individuals spent in the same subgroup with changes in d_th_ and t_th_ ; (d), (e), (f): Changes in the number of fusion events per month with changes in d_th_ and t_th_. Solid lines represent the predictions from the GAMM fitted to the original data and dashed lines represent 95% confidence intervals. The colors of line correspond to the different value of t_th_ (red: t_th_ = 2; green: t_th_ = 3; blue: t_th_ = 4; purple: t_th_ = 5; orange: t_th_ = 6).


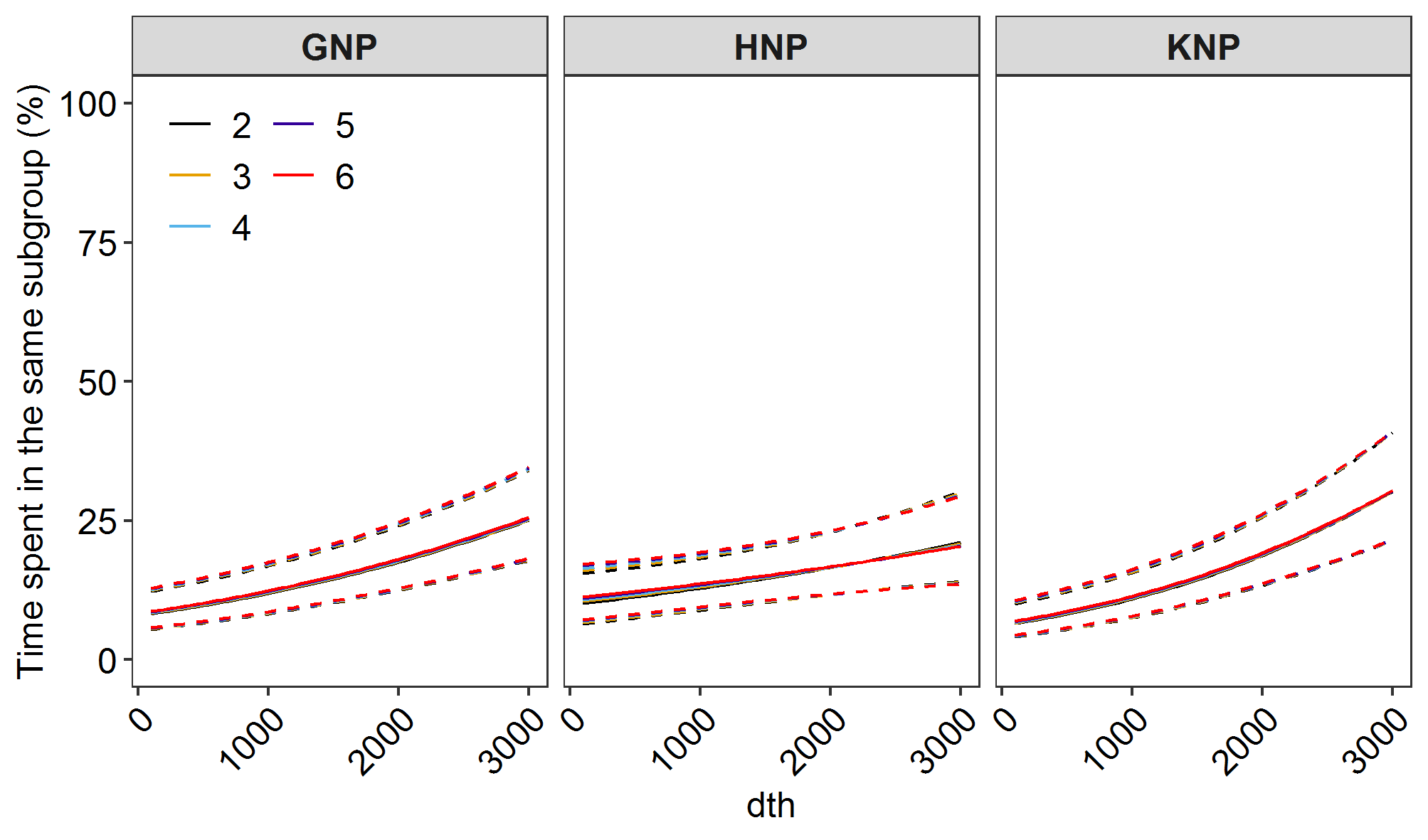

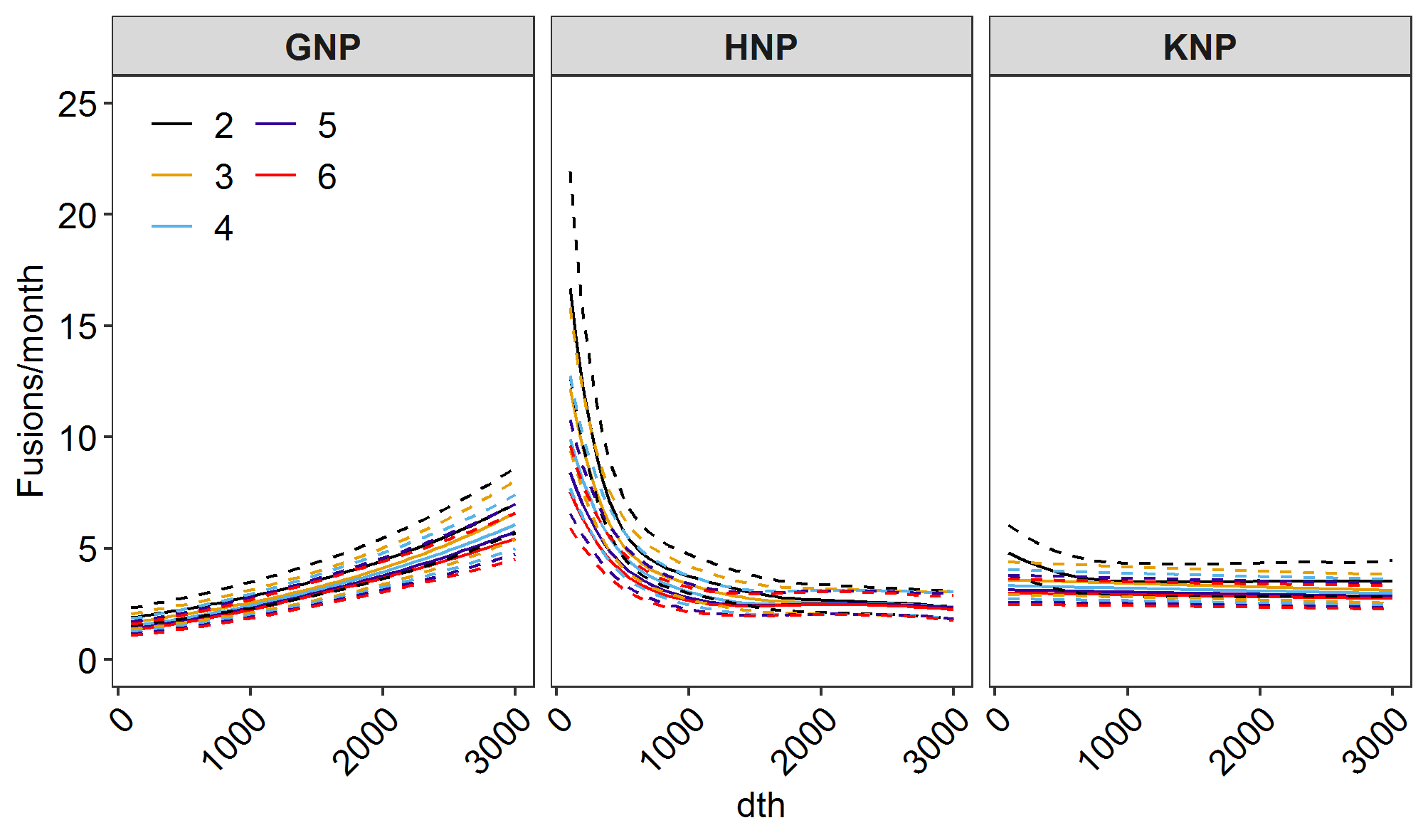


(a)

(b)

(c)

(d)

(e)

(f)

**4. Parameter estimates of most parsimonious models.**

**TABLE S3** Parameter estimates for the most parsimonious models presented in Table 1 in the main text. Estimates are reported with standard errors (s.e.) and 95% confidence intervals (95% CI). Explanatory variables varied by model but included HRO, season, site, distance to water and vegetation class. The standard deviations (SD) of random effects (dyad identity for analysis 1 and dyad identity nested within group identity for other analyses) is also given.

| Parameter | | Estimate | s.e. | 95% CI (lower; upper) |
| --- | --- | --- | --- | --- |
| 1. *Relationship between proportion of time spent in the same subgroup and home-range overlap* | | | | |
|  | Intercept [GNP, Dry] | -4.01 | 0.12 | (-4.26; -3.77) |
|  | Season [Wet] | -0.31 | 0.08 | (-0.46; -0.15) |
|  | Site [HNP] | 0.50 | 0.18 | (0.14; 0.85) |
|  | Site [KNP] | -0.03 | 0.15 | (-0.33; 0.27) |
|  | Site [HNP]: Season [Wet] | 0.36 | 0.16 | (0.04; 0.67) |
|  | Site [KNP]: Season [Wet] | 0.44 | 0.13 | (0.17; 0.70) |
|  | SD of random effect  Dyads | 0.36 |  |  |
| 1. *Seasonal changes in home range overlap* | | | | |
|  | Intercept | -0.001 | 0.038 | (-0.07; 0.07) |
|  | SD of random effect  Dyads:Herd  Herd | 0.00  0.08 |  |  |
| 1. *Seasonal changes in proportion of time spent in the same subgroup* | | | | |
|  | Intercept | 1.12 | 6.37 | (-11.37; 13.60) |
|  | SD of random effect  Dyads:Herd  Herd | 12.17  11.35 |  |  |
| 1. *Number of fusion events* | | | | |
|  | Intercept [Dry] | -0.65 | 0.22 | (-1.09; -0.21) |
|  | HRO | 2.84 | 0.26 | (2.33; 3.35) |
|  | Season [Wet] | 0.58 | 0.09 | (0.41; 0.75) |
|  | SD of random effect  Dyads:Herd  Herd | 0.34  0.00 |  |  |
| 1. *Duration of periods in the same subgroup* | | | | |
|  | Intercept [GNP, Dry] | 3.50 | 0.12 | (3.27; 3.73) |
|  | Season [Wet] | -0.56 | 0.07 | (-0.71; -0.41) |
|  | Site [HNP] | 0.84 | 0.17 | (0.50; 1.18) |
|  | Site [KNP] | 0.20 | 0.17 | (-0.13; 0.54) |
|  | SD of random effect  Dyads:Herd  Herd | 0.43  0.00 |  |  |
| 1. *Duration of periods in a different subgroup* | | | | |
|  | Intercept [GNP, Dry] | 7.01 | 0.35 | (6.32; 7.70) |
|  | HRO | -3.44 | 0.44 | (-4.30; -2.58) |
|  | Season  [Wet] | -0.61 | 0.07 | (-0.75; -0.46) |
|  | Site [HNP] | -0.51 | 0.15 | (-0.80; -0.21) |
|  | Site [KNP] | -0.43 | 0.15 | (-0.72; -0.14) |
|  | SD of random effect  Dyads:Herd  Herd | 0.33  0.00 |  |  |
| 1. *Occurrence of fusion events during the diel cycle* | | | | |
|  | Intercept | -1.15 | 0.10 | (-1.35; -0.96) |
|  | SD of random effect  Dyads:Herd  Herd | 0.33  0.19 |  |  |
| 1. *Occurrence of fission events during the diel cycle* | | | | |
|  | Intercept | -1.15 | 0.10 | (-1.35; -0.95) |
|  | SD of random effect  Dyads:Herd  Herd | 0.32  0.20 |  |  |
| 1. *Probability of fusion (vs. fission) in relation to vegetation class in wet season* | | | | |
|  | Intercept [Grassland] | 0.43 | 0.10 | (0.24; 0.61) |
|  | Vegetation [Bushland] | -0.49 | 0.11 | (-0.70; -0.28) |
|  | Vegetation [Woodland] | -0.52 | 0.12 | (-0.76; -0.29) |
|  | SD of random effect  Dyads:Herd  Herd | 0.00  0.00 |  |  |
| 1. *Probability of being in the same subgroup (vs. different subgroup) in relation to vegetation class in wet season* | | | | |
|  | Intercept [Grassland, GNP] | -0.61 | 0.24 | (-1.08; -0.13) |
|  | Vegetation [Bushland] | -0.06 | 0.01 | (-0.08; -0.04) |
|  | Vegetation [Woodland] | -0.14 | 0.01 | (-0.16; -0.12) |
|  | Site [HNP] | 0.55 | 0.35 | (-0.13; 1.23) |
|  | Site [KNP] | 0.70 | 0.33 | (0.05; 1.35) |
|  | Vegetation [Bushland]: Site [HNP] | 0.13 | 0.01 | (0.11; 0.15) |
|  | Vegetation [Woodland]: Site [HNP] | 0.13 | 0.01 | (0.10; 0.16) |
|  | Vegetation [Bushland]: Site [KNP] | -0.42 | 0.02 | (-0.45; -0.38) |
|  | Vegetation [Woodland]: Site [KNP] | -0.02 | 0.02 | (-0.06; 0.02) |
|  | SD of random effect  Dyads:Herd  Herd | 0.33  0.32 |  |  |
| 1. *Probability of fusion (vs. fission) in relation to distance to water and vegetation class in dry season* | | | | |
|  | Intercept [Grassland, GNP] | 1.29 | 0.29 | (0.73; 1.85) |
|  | Vegetation [Bushland] | -1.38 | 0.33 | (-2.02; -0.73) |
|  | Vegetation [Woodland] | -1.67 | 0.66 | (-2.96; -0.37) |
|  | Site [HNP] | 1.55 | 1.17 | (-0.75; 3.85) |
|  | Site [KNP] | -0.14 | 0.53 | (-1.17; 0.90) |
|  | Distance to water | -0.20 | 0.08 | (-0.36; -0.05) |
|  | Vegetation [Bushland]: Site [HNP] | -1.07 | 1.47 | (-3.95; 1.81) |
|  | Vegetation [Woodland]: Site [HNP] | 1.19 | 1.63 | (-2.00; 4.38) |
|  | Vegetation [Bushland]: Site [KNP] | 0.13 | 0.67 | (-1.19; 1.45) |
|  | Vegetation [Woodland]: Site [KNP] | 0.37 | 0.88 | (-1.34; 2.09) |
|  | Vegetation [Bushland]: Distance to water | 0.18 | 0.09 | (0.01; 0.34) |
|  | Vegetation [Woodland]: Distance to water | 0.23 | 0.11 | (0.01; 0.45) |
|  | Site [HNP]: Distance to water | -1.45 | 1.00 | (-3.41; 0.51) |
|  | Site [KNP]: Distance to water | -0.48 | 0.37 | (-1.21; 0.24) |
|  | Vegetation [Bushland]: Site [HNP]: Distance to water | 1.73 | 1.16 | (-0.54; 4.00) |
|  | Vegetation [Woodland]: Site [HNP]: Distance to water | -0.95 | 1.25 | (-3.40; 1.50) |
|  | Vegetation [Bushland]: Site [KNP]: Distance to water | 0.38 | 0.42 | (-0.43; 1.20) |
|  | Vegetation [Woodland]: Site [KNP]: Distance to water | 0.63 | 0.43 | (-0.20; 1.47) |
|  | SD of random effect  Dyads:Herd  Herd | 0.00  0.00 |  |  |
| 1. *Probability of being in the same subgroup (vs. different subgroup) in relation to distance to water and vegetation class in dry season* | | | | |
|  | Intercept [Grassland, GNP] | -0.07 | 0.23 | (-0.51; 0.38) |
|  | Vegetation [Bushland] | -0.10 | 0.01 | (-0.12; -0.07) |
|  | Vegetation [Woodland] | -0.08 | 0.02 | (0.04; 0.11) |
|  | Site [HNP] | 0.20 | 0.44 | (-0.66; 1.06) |
|  | Site [KNP] | 0.29 | 0.38 | (-0.46; 1.04) |
|  | Distance to water | -0.06 | 0.00 | (-0.07; -0.06) |
|  | Vegetation [Bushland]: Site [HNP] | 0.18 | 0.03 | (0.11; 0.24) |
|  | Vegetation [Woodland]: Site [HNP] | 0.03 | 0.03 | (-0.04; 0.09) |
|  | Vegetation [Bushland]: Site [KNP] | -0.24 | 0.03 | (-0.30; -0.18) |
|  | Vegetation [Woodland]: Site [KNP] | -0.43 | 0.03 | (-0.49; -0.36) |
|  | Vegetation [Bushland]: Distance to water | 0.00 | 0.00 | (0.00; 0.01) |
|  | Vegetation [Woodland]: Distance to water | -0.02 | 0.00 | (-0.02; -0.01) |
|  | Site [HNP]: Distance to water | -0.11 | 0.02 | (-0.14; -0.07) |
|  | Site [KNP]: Distance to water | -0.22 | 0.01 | (-0.25; -0.19) |
|  | Vegetation [Bushland]: Site [HNP]: Distance to water | 0.10 | 0.02 | (0.06; 0.14) |
|  | Vegetation [Woodland]: Site [HNP]: Distance to water | 0.00 | 0.02 | (-0.04; 0.04) |
|  | Vegetation [Bushland]: Site [KNP]: Distance to water | 0.17 | 0.02 | (0.14; 0.20) |
|  | Vegetation [Woodland]: Site [KNP]: Distance to water | 0.15 | 0.02 | (0.12; 0.18) |
|  | SD of random effect  Dyads:Herd  Herd | 0.56  0.25 |  |  |
